# Dissemination of the *Acinetobacter baumannii* isolates belonging to global clone 2 containing AbGRI resistance islands in a referral hospital

**DOI:** 10.1128/spectrum.05373-22

**Published:** 2023-08-28

**Authors:** Ghazal Naderi, Mahla Asadian, Arash Seifi, Sedigheh Ghourchian, Malihe Talebi, Mohammad Rahbar, Alireza Abdollahi, Masoumeh Douraghi

**Affiliations:** 1 Division of Microbiology, Department of Pathobiology, School of Public Health, Tehran University of Medical Sciences, Tehran, Iran; 2 Department of Infectious Diseases, Faculty of Medicine, Tehran University of Medical Sciences, Tehran, Iran; 3 Department of Microbiology, School of Medicine, Iran University of Medical Sciences, Tehran, Iran; 4 Department of Microbiology, Iranian Reference Health Laboratory Research Center, Ministry of Health and Medical Education, Tehran, Iran; 5 Department of Pathology, Imam Hospital Complex, Tehran University of Medical Sciences, Tehran, Iran; University of Manitoba, Winnipeg, Manitoba, Canada

**Keywords:** *Acinetobacter baumannii* genomic resistance island, global clone 2, newer antibiotics, older antibiotics, PCR mapping

## Abstract

*Acinetobacter baumannii* strains belonging to global clone 2 (GC2) contain resistance islands (AbGRIs), which are composed of genes conferring resistance to older and newer antibiotics. Here, to locate these genes in AbGRIs, the GC2 strains from Tehran, Iran were examined. Among the 170 *A*. *baumannii*, 90 isolates were identified as GC2. Of the genes that confer resistance to older antibiotics, *tetA(B*), *tetR(B)* (tetracyclines), *strA*, and *strB* (aminoglycosides) were located in AbGRI1 of 65 GC2 isolates (72.2%). Of the other aminoglycosides, the *aphA1b* was located in AbGRI2-12b (63.6%), AbGRI2-12a (21.2%), or AbGRI2-1 (15.1%). The *aacC1* and *aadA1* genes were co-located within AbGRI2-1 (5.5%). The *armA* was located in AbGRI3-4 (77.7%) and AbGRI3_ABI221_ (22.2%). Of sulfonamides, the *sul1* was located within AbGRI2-1 (5.5%). Of beta-lactams, the *bla_TEM_* was located in AbGRI2-12b (42%), AbGRI2-12a (14%), AbGRI2-1 (10%), or AbGRI2_ABI257_ (34%). The *oxa23* gene conferring resistance to newer antibiotics (carbapenems) was located in AbaR4 (81.1%); of them, the AbaR4 was located within AbGRI1 in 45.2% of the isolates. This study showed that the GC2 isolates, which contained at least one AbGRI, disseminate in the hospital. Hence, it is likely that the AbGRIs play a significant role in conferring resistance to older and newer antibiotics in GC2 isolates from Iran.

**IMPORTANCE:**

The majority of *Acinetobacter baumannii* isolates that are resistant to multiple antibiotics belong to one of the two major global clones, namely global clone 1 (GC1) and global clone 2 (GC2). The resistance islands, which contain variable assortments of transposons, integrons, and specific resistance genes, have been characterized in the genome of these GCs. In GC2 *A. baumannii,* the chromosomally located *A. baumannii* genomic resistance islands (AbGRIs) carry the genes conferring resistance to older and newer antibiotics. In this context, we tested whether GC2 isolates collected from a referral hospital carry the AbGRIs containing these genes. This study provided evidence for the circulation of the GC2 *A. baumannii* strains harboring AbGRI resistance islands between different wards of a referral hospital.

## INTRODUCTION

*Acinetobacter baumannii* is regarded as a global threat due to its capability of developing resistance to most of the currently available antimicrobial agents. In addition to the intrinsic resistance to many antibiotics, this organism also has a remarkable capacity to acquire antibiotic resistance determinants (1). A number of resistance islands (RIs) have been identified among multidrug-resistant isolates of *A. baumannii*, mainly in the isolates belonging to two major global clones 1 and 2 (GC1 and GC2) (2, 3). The RIs, which differ in structure in GC1 and GC2 isolates, are composed of complex transposons harboring mobile genetic elements [such as insertion sequences (IS) and integrons] and various antibiotic resistance genes. *A. baumannii* resistance islands (AbaRs) are mainly found in GC1 isolates, and *A. baumannii* genomic resistance islands (AbGRIs) are mainly found in GC2 isolates (3). The AbGRI1 (4), AbGRI2 (5, 6), and AbGRI3 (7) include the major RIs that are identified among GC2 isolates. The AbGRIs include some or all of the genes conferring resistance to older antibiotics, including *tetA*(B), *tetR*(B) (tetracyclines), *aacC1*, *aacA4, aphA1b*, *aadA1*, *strA*, *strB*, *armA* (aminoglycosides), *sul1, sul2* (sulfonamides), *bla_TEM_* (beta-lactams), and the genes conferring resistance to newer antibiotics, including *oxa23* (carbapenems) (4 – 7). In GC2 isolates, *oxa23* gene is often found in Tn*2006* alone or in AbaR4 (Tn*2006* inserted in Tn*6022*), and AbaR4Δ1 (Tn*2006* inserted in Tn*6022Δ1*) as components of the AbGRI1 and in additional positions in the chromosome at locations outside the AbGRIs or on plasmids (8, 9).

The high levels of resistance to older and newer antibiotics were reported in the previous study from Iran (10). However, little is known regarding the role of AbGRIs in conferring resistance to older and newer antibiotics from Iran and other Middle East countries (11). Here, we aimed to locate these genes in AbGRIs in GC2 strains from Tehran, Iran.

## RESULTS

### Identification of GC2 isolates and antibiotic resistance profiles

Of the isolates examined, 90 isolates belonged to GC2. All isolates were resistant to carbapenems (imipenem, meropenem, and doripenem); however, resistance to older antibiotics, including tetracyclines, aminoglycosides, and sulfonamides, varied among the isolates. The antibiotic resistance profile of the isolates is shown in Table 1. The results of disk diffusion for GC2 *A. baumannii* isolates are presented in Table S1.

**TABLE 1:** The patterns identified in the isolates, according to the type of AbGRI1, AbGRI2, and AbGRI3 resistance islands carried by the isolates^*i*^

Pattern	AbGRI1, AbGRI2, AbGRI3	Isolate	MLST (Pasteur)	Antibiotic resistance profile^*a*^	Isolation date	Ward	Source
1	No AbGRI1, AbGRI2_ABI257_, AbGRI3-4	**ABI270**	2	CASmSpSuTcKmGmAkTmNe^*h*^	December 2019	ICU 10	Blood
		***ABI151^b^***	2	CASmSpSuTcKmGmAkTmNe	January 2019	ICU 1	BAL
		ABI179	–^*j*^	CASmSpSuTcKmGmAkTmNe	January 2019	Ward 1	Wound
		ABI223	–	CASmSpSuTcKmGmAkTmNe	July 2019	ICU 3	Trachea
		ABI271	–	CASmSpSuTcKmGmAkTmNe	December 2019	ICU 4	Blood
		ABI281	–	CASmSpSuTcKmGmAkTmNe	January 2020	ICU 10	Sputum
2	No AbGRI1, AbGRI2-12b, AbGRI3-4	**ABI169**	2	CASmSpSuTcKmNmGmAkTmNe	December 2018	ICU 5	Blood
		ABI191	–	CASmSpSuTcKmNmGmAkTmNe	February 2019	Ward 2	Blood
		ABI198	–	CASmSpSuTcKmNmGmAkTmNe	June 2019	ICU 6	Trachea
		ABI222	–	CASmSpSuTcKmNmGmAkTmNe	July 2019	ICU 7	Discharges
		ABI249	–	CASmSpSuTcKmNmGmAkTmNe	October 2019	ICU 1	Discharges
3	No AbGRI1, AbGRI2-12a, AbGRI3-4	**ABI199**	2	CASmSpSuKmNmGmAkTmNe	June 2019	Ward 4	Pleural effusion
		ABI259	–	CASmSpSuKmNmGmAkTmNe	November 2019	ICU 5	Sputum
		ABI194	–	CASmSpSuTcKmNmGmAkTmNe	June 2019	Ward 3	Ascites
		ABI254	–	CASmSpSuTcKmNmGmAkTmNe	October 2019	ICU 8	Trachea
4	No AbGRI1, AbGRI2_ABI257,_ AbGRI3_ABI221_	**ABI277**	2	CASmSpSuTcKmGmAkTmNe	January 2020	ICU 10	Trachea
5	No AbGRI1, No AbGRI2, AbGRI3-4	**ABI242**	2	CASmSpSuTcKmGmAkTmNe	September 2019	ICU 10	Trachea
		**ABI284^*c*^ **	113	CASmSpSuKmGmAkTmNe	January 2020	ICU 10	Trachea
		ABI159	–	CASmSpSuTcKmNmGmAkTmNe	December 2018	Ward 5	CSF
		ABI230	–	CASmSpSuTcKmGmAkTmNe	August 2019	Ward 3	Trachea
		ABI171	–	CASmSpSuTcKmGmAkTmNe	December 2018	ICU 7	Blood
		ABI185	–	CASmSpSuTcKmGmAkTmNe	January 2019	ICU 9	Trachea
		ABI190	–	CASmSpSuTcKmGmAkTmNe	January 2019	ICU 1	Trachea
		ABI204	–	CASmSpSuKmNmGmAkTmNe	July 2019	ICU 10	Trachea
6	No AbGRI1, AbGRI2-12b, No AbGRI3	**ABI229**	10	CASmSpSuTcKmNmTm	August 2019	Ward 7	Trachea
7	Group 4, no AbGRI2, AbGRI3-4	**ABI215**	2	CASmSpTcKmNmGmAkTmNe	July 2019	ICU 10	Blood
		ABI201	–	CASmSpTcKmNmGmAkTmNe	July 2019	ICU 2	Ascites
		ABI206	–	CASmSpTcKmNmGmAkTmNe	July 2019	ICU 9	Trachea
		ABI233	–	CASmSpTcKmGmAkTmNe	August 2019	Ward 9	Blood
		ABI272	–	CASmSpTcKmGmAkTmNe	January 2020	Ward 8	Blood
8	Group 4, no AbGRI2, AbGRI3_ABI221_	***ABI203^d^***	2	CASmSpTcKmNmGmAkTmNe	July 2019	Ward 10	CSF
		ABI251	–	CASmSpTcKmNmGmAkTmNe	October 2019	Ward 11	Trachea
9	Group 1, no AbGRI2, AbGRI3_ABI221_	**ABI239**	2	CASmSpSuTcKmNmGmAkTmNe	September 2019	ICU 8	Trachea
		ABI205	–	CASmSpSuTcKmNmGmAkTmNe	July 2019	ICU 10	Trachea
		ABI216	–	CASmSpSuTcKmNmGmAkTmNe	July 2019	ICU 8	Trachea
		ABI255	–	CASmSpSuTcKmGmAkTmNe	October 2019	Ward 12	Sputum
		ABI260	–	CASmSpSuTcKmGmAkTmNe	November 2019	ICU 9	Catheter
		ABI265	–	CASmSpSuTcKmGmAkTmNe	November 2019	Ward 13	Catheter
10	Group 2, no AbGRI2, AbGRI3_ABI221_	**ABI219**	2	CASmSpSuTcKmNmGmAkTmNe	July 2019	Ward 14	Synovial fluid
11	Group 1, no AbGRI2, AbGRI3-4	**ABI231**	2	CASmSpSuTcKmGmAkTmNe	August 2019	Ward 7	Wound
		**ABI250**	2	CASmSpSuTcKmGmAkTmNe	October 2019	Ward 5	Sputum
		**ABI001**	2	CASmSpSuTcKmGmAkTmNe	November 2011	ICU 10	Blood
		ABI261	–	CASmSpSuTcKmGmAkTmNe	November 2019	ICU 9	Discharges
		ABI020	–	CASmSpSuTcKmGmAkTmNe	December 2011	ICU 10	Trachea
		ABI227	–	CASmSpSuTcKmNmGmAkTmNe	August 2019	ICU 2	Trachea
		ABI034	–	CASmSpSuTcKmNmGmAkTmNe	January 2012	ICU 10	Wound
12	Group 2, no AbGRI2, no AbGRI3	***ABI154^e^***	2	CASmSpSuTcNm	December 2018	ICU 2	Discharges
		**ABI024**	2	CASmSpSuTcAk	ABI024	Ward 5	Trachea
		ABI002	–	CASmSpSuTcAk	November 2011	ICU 10	Blood
		ABI052	–	CASmSpSuTcAk	June 2012	NR	NR
		ABI167	–	CASmSpSuTcNm	December 2018	ICU 1	Blood
		ABI181	–	CASmSpSuTcNm	January 2019	Ward 15	Wound
		ABI163	–	CASmSpSuTcNmAk	December 2018	ICU 5	Trachea
		ABI275	–	CASmSpSuTcNmAk	January 2020	ICU 10	Trachea
		ABI157	–	CASmSpSuTcAkNe	December 2018	ICU 10	Trachea
13	Group 4, no AbGRI2, no AbGRI3	**ABI196**	2	CASmSpTcNmAk	June 2019	Ward 16	Sputum
14	Group 1, no AbGRI2, no AbGRI3	**ABI220**	2	CASmSpSuTcAk	July 2019	Ward 17	Pericardial effusion
15	Group 4, AbGRI2-1, no AbGRI3	***ABI184^f^***	2	CASmSpSuTcKmNmGmAk	January 2019	Ward 18	Discharges
		ABI195	–	CASmSpSuTcKmNmGmAk	June 2019	ICU 1	Trachea
16	Group 3, AbGRI2-1, no AbGRI3	***ABI188^g^***	2	CASmSpSuTcKmNmGmAk	January 2019	ICU 10	Discharges
		ABI269	–	CASmSpSuTcKmNmGmAk	November 2019	ICU 10	Blood
		ABI045	–	CASmSpSuTcKmNmGmAkTm	May 2012	NR	Wound
17	Group 2, AbGRI2-12b, no AbGRI3	**ABI007**	2	CASmSpSuTcKmNm	November 2011	ICU 10	Blood
18	Group 1, AbGRI2-12b, AbGRI3-4	**ABI005**	2	CASmSpSuTcKmGmAkTmNe	November 2011	Ward 13	Ascites
		**ABI252**	2	CASmSpSuTcKmNmGmAkTmNe	October 2019	ICU 5	Trachea
		ABI143	–	CASmSpSuTcKmNmGmAkTmNe	December 2018	ICU 3	Trachea
		ABI192	–	CASmSpSuTcKmNmGmAkTmNe	January 2019	ICU 10	Blood
		ABI014	–	CASmSpSuTcKmNmGmAkTmNe	December 2011	NR	Trachea
		ABI026	–	CASmSpSuTcKmNmGmAkTmNe	January 2012	Ward 5	NR
		ABI028	–	CASmSpSuTcKmNmGmAkTmNe	April 2012	NR	Trachea
		ABI046	–	CASmSpSuTcKmNmGmAkTmNe	May 2012	NR	Wound
		ABI076	–	CASmSpSuTcKmNmGmAkTmNe	September 2012	NR	NR
		ABI248	–	CASmSpSuTcKmGmAkTmNe	October 2019	ICU 10	Trachea
19	Group 1, AbGRI2_ABI257,_ AbGRI3-4	**ABI268**	2	CASmSpSuTcKmGmAkTmNe	November 2019	ICU 9	Trachea
		ABI262	–	CASmSpSuTcKmGmAkTmNe	November 2019	Ward 19	Ascites
		ABI286	–	CASmSpSuTcKmGmAkTmNe	January 2020	Ward 20	Discharges
		ABI266	–	CASmSpSuTcKmNmGmAkTmNe	November 2019	ICU 10	Trachea
20	Group 2, AbGRI2-12b, AbGRI3-4	**ABI193**	2	CASmSpSuTcKmNmGmAkTmNe	January 2019	ICU 10	Blood
		ABI166	–	CASmSpSuTcKmNmGmAkTmNe	December 2018	ICU 4	Trachea
		ABI189	–	CASmSpSuTcKmNmGmAkTmNe	January 2019	ICU 4	Trachea
21	Group 4, AbGRI2-12b, AbGRI3-4	**ABI207**	2	CASmSpTcKmNmGmAkTmNe	July 2019	Ward 21	Wound
22	Group 1, AbGRI2_ABI257,_ AbGRI3_ABI221_	**ABI221**	2	CASmSpSuTcKmGmAkTmNe	July 2019	Ward 2	Wound
		**ABI257**	2	CASmSpSuTcKmGmAkTmNe	November 2019	Ward 9	Ascites
		ABI243	–	CASmSpSuTcKmGmAkTmNe	September 2019	ICU 10	Trachea
		ABI258	–	CASmSpSuTcKmGmAkTmNe	November 2019	Ward 4	Discharges
		ABI263	–	CASmSpSuTcKmGmAkTmNe	November 2019	ICU 9	Blood
		ABI283	–	CASmSpSuTcKmGmAkTmNe	January 2020	Ward 21	Wound
23	Group 1, AbGRI2-12a, AbGRI3-4	**ABI004**	2	CASmSpSuTcKmNmGmAkTmNe	November 2011	Ward 21	Catheter
		ABI256	–	CASmSpSuTcKmNmGmAkTmNe	November 2019	Ward 6	Wound
		ABI273	–	CASmSpSuTcKmNmGmAkTmNe	January 2020	Ward 9	Blood

^*a*^ The profile is relevant to the genes conferring resistance to older and newer antibiotics, which are located on AbGRI1, AbGRI2, and AbGRI3 resistance islands.

^*b*^ Oxf-ST2868 (*gltA*-1, *gyrB*-38, *gdhB*-3, *recA*-2, *cpn60*-2, *gpi*-107, *rpoD*-3).

^*c*^ The chromosomal *comM* gene was found to be uninterrupted, and the J1 and J2 junction PCRs failed to amplify in this isolate.

^*d*^ Oxf-ST1624 (*gltA*-1, *gyrB*-50, *gdhB*-3, *recA*-2, *cpn60*-2, *gpi*-94, *rpoD*-3).

^*e*^ Oxf-ST452 (*gltA*-1, *gyrB*-12, *gdhB*-3, *recA*-2, *cpn60*-2, *gpi*-110, *rpoD*-3).

^*f*^ Oxf--ST2869 (*gltA*-1, *gyrB*-12, *gdhB*-3, *recA*-2, *cpn60*-2, *gpi*-156, *rpoD*-3).

^*g*^ Oxf-ST1624 (*gltA*-1, *gyrB*-50, *gdhB*-3, *recA*-2, *cpn60*-2, *gpi*-94, *rpoD*-3).

^*h*^ CA: carbapenems, including imipenem, meropenem, and doripenem (all isolates were resistant to carbapenems), Sm: streptomycin, Sp: spectinomycin, Su: sulfamethoxazole, Tc: tetracycline, Km: kanamycin, Nm: neomycin, Gm: gentamicin, AK: amikacin, Tm: tobramycin, Ne: netilmicin. BAL, bronchoalveolar lavage; CSF, cerebrospinal fluid; MLST, multi-locus sequence typing; NR, not recorded.

^*i*^ The representative isolates of each pattern are in bold, which their ST was determined. The italicized isolates are the isolates for which MLST using both the Pasteur and Oxford schemes was performed. The underlined isolates are the isolates for which MLST using only the Pasteur scheme was performed.

^*j*^ –, ST is not determined.

### The genes conferring resistance to older antibiotics carried by AbGRI resistance islands

**Fig 1:**
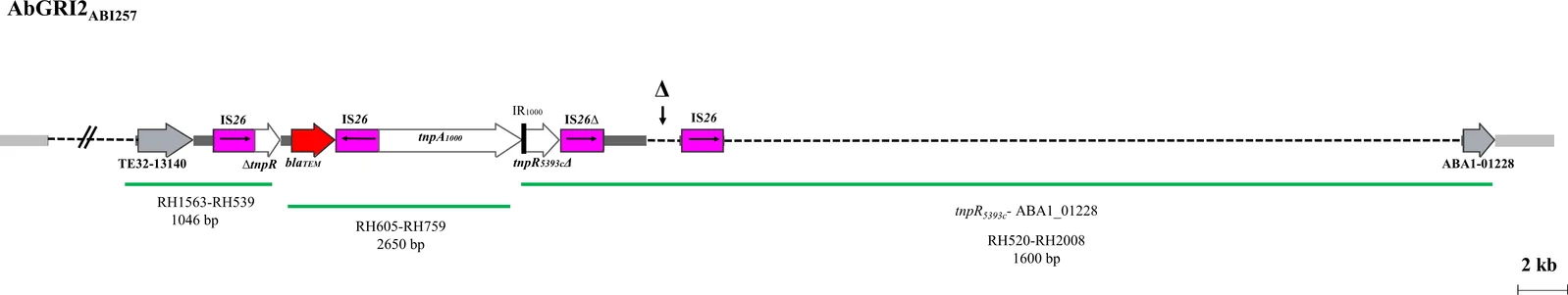
Schematic of the AbGRI2_ABI257_. The central dark gray line represents the resistance island. The light gray line indicates the adjacent chromosomal sequence. Arrows indicate extent and orientation of genes and open reading frames. The resistance genes are red with the name below. Boxes represent IS, and the internal arrows indicate the transposase. The vertical arrows indicate the location of deletion (Δ) found in the AbGRI2_ABI257_ in this study. Dashed lines in AbGRI2_ABI257_ represent the extent of deletions relative to AbGRI2-12. The primers used to link sequences are indicated beneath each green line, along with the predicted amplicon size. The figure is adapted from reference (8) with permission from Dr. Blackwell.

As expected, the typical AbGRI1 genes, including *tetA(B), tetR(B*), *strA*, and *strB,* which confer resistance to older antibiotics, were co-located in the 65 GC2 isolates (72.2%) carrying AbGRI1. Of 90 GC2 isolates, 65 isolates did not contain an intact *comM,* while both the J1 and J2 junctions were present and orf4b (shares 88% identity with orf4 in Tn*6022*) was adjacent to *comM* gene*,* indicating that they contained AbGRI1 resistance island (Table S2). The *aphA1b* (aminoglycosides) was carried by Tn*6020*; of them, all were located in AbGRI2-12b (63.6%), AbGRI2-12a (21.2%), or AbGRI2-1 (15.1%) in 33 GC2 isolates (36.6%). The *sul1* (sulfonamides), *aacC1*, and *aadA1* (aminoglycosides) genes were located in a class 1 integron within AbGRI2-1 in five GC2 isolates (5.5%). The *bla_TEM_* (beta-lactams) was located in AbGRI2-12b (42%), AbGRI2-12a (14%), AbGRI2-1 (10%), or a structure that is similar to AbGRI2-12 but has lost a segment from the right-hand side of the island (34%) in 50 GC2 isolates (55.5%) (Table S3). In the isolates which appear to lose a segment from the right-hand side of the AbGRI2, a PCR that was performed to link the *tnpR*5393c to the right end of the island produced a 1.7-kb amplicon. The sequencing of this product revealed that likely a deletion at the right end has removed a segment from the right-hand side of the island; hence, this island was named AbGRI2_ABI257_. The genetic structure of the AbGRI2_ABI257_ is shown in Fig. 1. The *armA* gene (aminoglycosides) was located in AbGRI3-4 (77.7%), or a structure that is similar to AbGRI3-4 but has lost a segment from the left-hand side of the island (22.2%) in 72 GC2 isolates (80%) (Table S3). In the isolates which appear to lose a segment from the left-hand side of the AbGRI3, a PCR linking the left end of the island to ISAba24 amplified a 3.8-kb amplicon. Sequencing of this amplicon revealed that these isolates had lost a segment from the left-hand side of the island; hence, this island was named AbGRI3_ABI221_ (12). In this study, the *aacA4* gene, which is associated with AbGRI3, was not detected in the GC2 isolates.

In the isolates found to carry AbGRI1, four groups were identified based on their backbone transposon and the presence of the *sul2* gene. The isolates in the first group contained both Tn*6022* and Tn*6022*Δ1 (with a specific deletion in respect to Tn*6022*); however, the isolates in groups 2 and 3 contained Tn*6022*Δ1 and Tn*6022*, respectively. While the *sul2* gene was present in the isolates in groups 1–4, it was absent in the isolates in group 4 (Table S4). In the isolates containing the *sul2* gene, the primers linking *sul2* to *comM* gene (RH928 and sul2F) failed to amplify a product of the predicted size of 16,826 bp, which is not surprising due to the predicted large amplicon size. Furthermore, since AbGRI1-1 and AbGRI1-6 are the only known AbGRI1s without the *sul2* gene, the mapping strategies for AbGRI1-1 and AbGRI1-6 were attempted for the isolates without the *sul2* gene. As all isolates were positive for the PCR used to link the *oxa23* to *tetA(B*), they were similar to AbGRI1-6; however, they did not contain Tn*6022*Δ1. Therefore, it was not possible to determine the genetic structure of AbGRI1 in both the isolates containing *sul2* and the isolates without this gene.

### The genes conferring resistance to newer antibiotics carried by AbGRI1 resistance islands

The *oxa23* gene (carbapenems), which confers resistance to newer antibiotics, was detected in all GC2 isolates. The *oxa23* gene was located in AbaR4 (81.1%); of them, 45.2% were positive for the PCR used to link the *oxa23* to *tetA(B*), indicating that the AbaR4 was located within AbGRI1 in these isolates (Table S2).

### Dissemination of the GC2 isolates containing AbGRI resistance islands in the hospital

**Fig 2:**
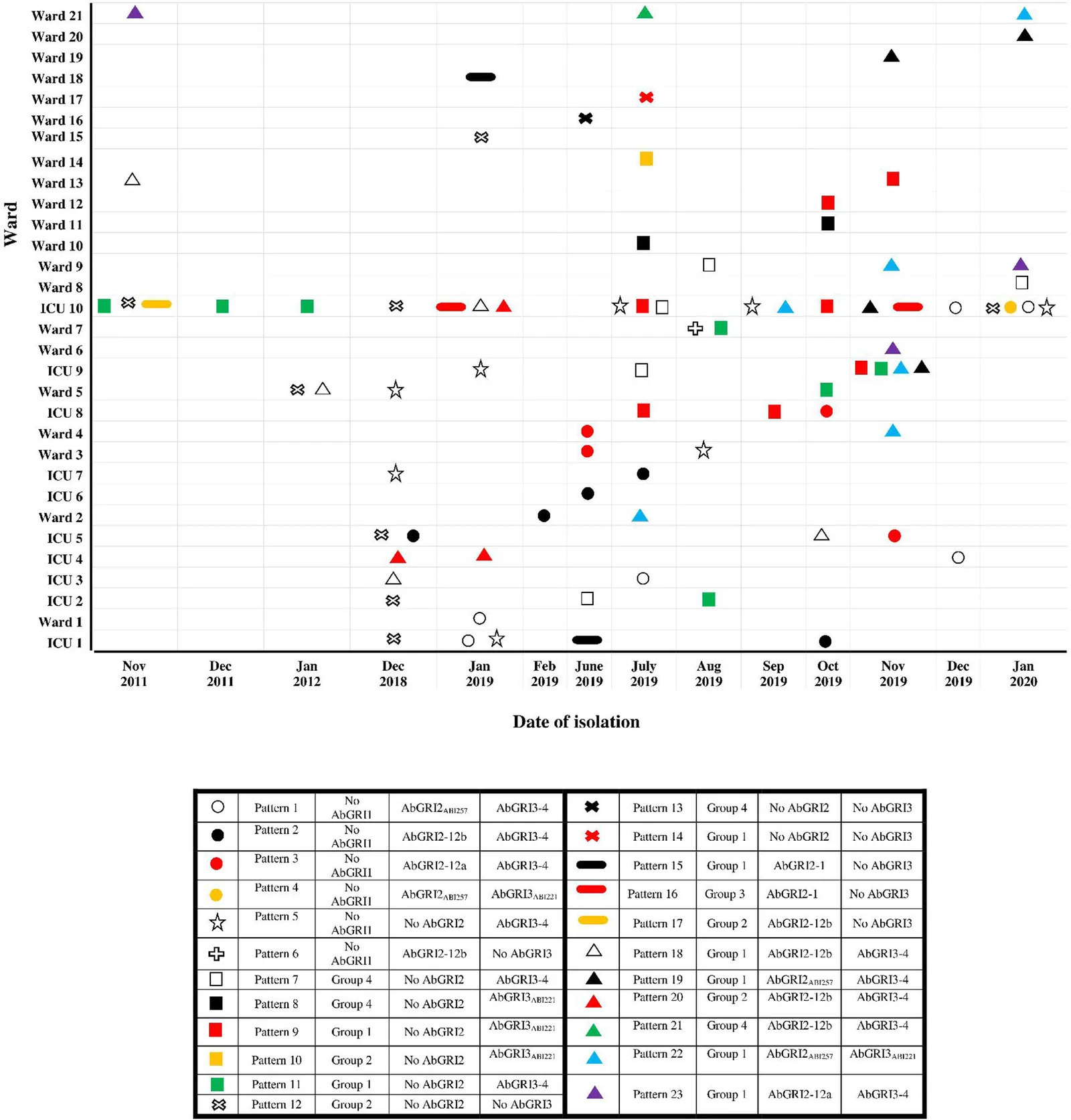
Schematic of the dissemination of GC2 isolates between hospital wards. The symbols indicate different patterns identified in the isolates (according to the type of AbGRIs carried by the isolates) as shown in the key below the figure.

Based on the type of AbGRI resistance islands carried by the isolates, 23 patterns were detected in the GC2 isolates (Table 1). Of the 23 patterns detected, 22 were identified in the set 2 isolates (collected during 2018–2020). In addition, six patterns were detected in set 1 isolates (collected during 2011–2012); of them, five patterns were shared between sets 1 and 2, and one pattern was only identified in set 1 isolates. The patterns including all three AbGRIs were the most frequent (6 patterns, in 27 isolates), followed by five patterns including both AbGRI1 and AbGRI3 (in 21 isolates), four patterns including both AbGRI2 and AbGRI3 (in 16 isolates), three patterns including only AbGRI1 (in 11 isolates), three patterns including both AbGRI1 and AbGRI2 (in six isolates), three patterns including only AbGRI2 (in one isolate), and a pattern including only AbGRI13 (in eight isolates). All but two selected GC2 isolates that were representatives of each pattern (according to the type of AbGRI resistance islands carried by the isolates) were ST2 (*cpn60*-2, *fusA*-2, *gltA*-2, *pyrG*-2, *recA*-2, *rplB*-2, *rpoB*-2). The remaining two isolates were ST10 (*cpn60*-1, *fusA*-3, *gltA*-2, *pyrG*-1, *recA*-4, *rplB*-4, *rpoB*-4) and ST113 (*cpn60*-3, *fusA*-3, *gltA*-3, *pyrG*-4, *recA*-7, *rplB*-4, *rpoB*-4), according to the Institut Pasteur scheme. Using the Oxford scheme, among five isolates, four different STs were detected, including ST1624 (*gltA*-1, *gyrB*-50, *gdhB*-3, *recA*-2, *cpn60*-2, *gpi*-94, *rpoD*-3) in two isolates, ST452 (*gltA*-1, *gyrB*-12, *gdhB*-3, *recA*-2, *cpn60*-2, *gpi*-110, *rpoD*-3) in one isolate, and two new STs (ST2868, ST2869) with the following allelic profiles: *cpn60*-1, *fusA*-38, *gltA*-3, *pyrG*-2, *recA*-2, *rplB*-107, *rpoB*-3 and *cpn60*-1, *fusA*-12, *gltA*-3, *pyrG*-2, *recA*-2, *rplB*-156, *rpoB*-3 (Table 1). ST1624, ST2868, and ST2869 were double locus variants of ST452. Furthermore, it was notable that most of the isolates with the same pattern according to the type of AbGRI resistance islands carried by the isolates contained the same antibiotic resistance profile (Table 1). Although most of the isolates belonged to the ST2 sequence type according to the Pasteur scheme, various antibiotic resistance profiles were identified. Two isolates that did not belong to ST2, according to the Pasteur scheme (ABI229 and ABI284, Table 1), had unique antibiotic resistance profiles. The patterns, which were described here based on the type of AbGRI resistance islands carried by the isolates, were identified in the isolates recovered from the patients admitted to different wards of the hospital, indicating their dissemination between the hospital wards (Fig. 2).

## DISCUSSION

*A. baumannii* has attracted significant attention due to the occurrence of strains that are resistant to almost all the available antimicrobial agents (1). The *A. baumannii* strains that are resistant to older antibiotics, including tetracycline, sulfonamides, and aminoglycosides, began to be noticed since the 1970s (13), and the strains that are resistant to newer antibiotics, such as carbapenems, were recognized in 1985 (14). In the previous studies, the genes conferring resistance to older and newer antibiotics have been found in GC2 isolates harboring AbGRIs (4 – 7); however, much less is known about them in the Middle East region, including Iran (11). Here, to locate the genes conferring resistance to older and newer antibiotics in AbGRIs, the GC2 strains from Tehran, Iran, were examined. Of the genes which confer resistance to older antibiotics, the *tetA(B), tetR(B)* (tetracycline), *strA*, and *strB* (aminoglycosides) genes were located in AbGRI1 in more than two-thirds of the GC2 isolates tested. In consistent with this finding, 82.4% of the isolates examined in South Korea carried AbGRI1-type resistance islands (9). In addition, all 62 GC2 isolates examined in Australia contained AbGRI1 resistance island (15). In 2013, the term AbGRI was proposed for the RIs that were found in GC2 isolates (6). What are currently called AbGRI1 variants were previously named as AbaR or Tn, or (incorrectly) as derivatives of AbaR4 in the studies conducted in the countries such as Lithuania (16), South Korea (17, 18), Latvia (19), and Australia (20). The AbGRI2 resistance islands, which usually contain some or all of the *aphA1b*, *aacC1*, *aadA1*, *bla*_TEM_, and *sul1* genes conferring resistance to antibiotics used clinically in the 1970s (5), were identified in the genomes of GC2 isolates in 2013 (6) and later, a number of variants were characterized in the isolates from Australia and Singapore and also in the sequences of published genomes (8, 15, 21). While only 15% of GC2 isolates from Singapore contained AbGRI2-12b (8), this island was detected in nearly half of the isolates examined in this study. The AbGRI2-1 harboring a class 1 integron was detected in 5.5% of the isolates tested in this study; however, it was present in 29% and 25% of GC2 isolates examined in Australia and Singapore, respectively (8, 15). Although Blackwell et al. found the *armA* in AbGRI3-4 in 46.7% of the GC2 isolates (7), this gene was located in AbGRI3-4 in more than two-thirds of the isolates examined here. In addition, a new structure of AbGRI3 resistance island was found in this study. The *bla_TEM_* gene, which confers resistance to beta-lactams, was mostly located in AbGRI2-12b (42%) and with lower frequency in AbGRI2-12a or AbGRI2-1. Furthermore, a new structure of AbGRI2 resistance island that carries *bla_TEM_* was found in this study (Fig. 1). The *sul1* gene which confers resistance to sulfonamides was located in a class 1 integron within AbGRI2-1 in only five GC2 isolates. Of the carbapenem-resistant GC2 isolates examined here, all carried the *oxa23* gene. The *oxa23* gene conferring resistance to newer antibiotics was located in AbaR4 (81.1%), which the AbaR4 was located within AbGRI1 in 45.2% of the isolates. In a study conducted in Singapore (8), the AbaR4 was located in AbGRI1 in 40% of the GC2 isolates. In consistent with this study, the AbaR4 was located in AbGRI1 in 45.2% of our isolates. In this study, the *sul2* gene was only absent in the isolates in group 4 (20%) (four groups were identified in the isolates to carry AbGRI1 based on their backbone transposon and the presence of the *sul2* gene). In consistent with this study, 13.84% of the isolates examined in Singapore did not contain the *sul2* gene (8). In the isolates containing the *sul2* gene in this study, the primers linking *sul2* to *comM* gene (RH928 and sul2F) failed to amplify a product of the predicted size of 16,826 bp, which is not surprising due to the predicted large amplicon size. Consequently, sequencing using long-read technology, such as PacBio or Oxford Nanopore, will be required to determine the genetic structure of AbGRI1 in these isolates.

It is noteworthy that in this study, two sets of isolates were recovered from the patients admitted to a single hospital at different time periods of 2011–2012 (set 1) and 2018–2020 (set 2). Hence, it was possible to compare these two sets of isolates in terms of the location of genes conferring resistance to older and newer antibiotics. Based on the presence or absence of AbGRI resistance islands in the GC2 isolates, 23 patterns were identified. Of the 23 patterns detected, 22 were identified in the isolates collected from 2018 to 2020. In addition, six patterns were detected in the isolates collected earlier during 2011–2012; of them, five patterns were shared between two sets of isolates. When using the Institut Pasteur scheme to perform multi-locus sequence typing (MLST) for the representative isolates of each pattern (according to the type of AbGRI resistance islands carried by the isolates), three STs were identified (Table 1), including ST2 (28/30, 93.3%), ST10 (1/30, 3.3%), and ST113 (1/30, 3.3%). It is demonstrated that ST2 is the most dominant ST sequence type in GC2 strains (22). Although the ST of isolates was mostly the same according to the Institut Pasteur scheme, several patterns were identified according to the type of AbGRI resistance islands carried by the isolates. These patterns were identified in the isolates recovered from the patients in the different wards of the hospital, indicating their dissemination between the hospital wards. It was found that the isolate belonging to ST10 (Institut Pasteur scheme) contains unique pattern based on both the type of AbGRI resistance islands carried by the isolates and also antibiotic resistance profile. In addition, the isolate belonging to ST113 (Institut Pasteur scheme), which was the only GC2 isolate with the uninterrupted chromosomal *comM* gene, contained a unique antibiotic resistance profile. As little intra-clonal variability has been observed using the Institut Pasteur MLST scheme, it was not possible to differentiate the circulating strains in different wards of the hospital based on this scheme (23). However, the isolates could be tracked according to the type of AbGRI resistance islands carried by the isolates. The Oxford MLST scheme is more capable to differentiate closely related isolates, and there has been much more intra-clonal diversity observed using this scheme (24). Using the Oxford scheme, four different STs were detected (ST1624, ST452, and two new STs (ST2868, ST2869) (Table 1). Most of this diversity is due to changes in the *gpi* allele, which is caused by capsule locus replacement and would cause a change in ST (25). As the STs of limited isolates were determined using the Oxford scheme in this study, the diverse STs that were observed were correlated with the diverse patterns identified based on the type of AbGRI resistance islands carried by the isolates. Although two isolates belonged to the same ST1624 according to the Oxford scheme, they belonged to the different patterns based on the type of AbGRI resistance islands carried by the isolates and also based on the antibiotic resistance profile.

This study provides evidence for the dissemination of GC2 isolates carrying AbGRI resistance islands in a referral hospital in Tehran, Iran. In fact, the features of AbGRI1, AbGRI2, and AbGRI3 resistance islands, alongside antibiotic resistance profile, were informative epidemiological markers to find the relationships between the isolates, which were distributed within the hospital. This study demonstrated that the strains carrying the AbGRI resistance islands are not restricted to a particular geographical region, and they are globally distributed. The results obtained in this study will underpin future studies of GC2 strains from the Middle Eastern countries.

## MATERIALS AND METHODS

### Bacterial isolates

A total of 170 non-repetitive *A. baumannii* isolates were collected from a single referral hospital in Tehran, Iran in different time periods of November 2011 to September 2012 (*n* = 45; set 1) and between December 2018 and January 2020 (*n* = 125; set 2), except for the 3 months interval in 2019. The ethics committee of Tehran University of Medical Sciences approved the study “IR. TUMS.SPH. REC.1397.291.” All patients have signed the informed consent for giving the required specimens for research. Parents/legal guardians provided written-informed consent for their children under the age of 18 years to participate in this study. All isolates were initially identified using conventional microbiological methods (26) and were further confirmed by the detection of *oxaAb* gene by PCR (27).

### Antibiotic susceptibility testing

Antibiotic susceptibility testing was performed by disk diffusion method using the following 27 antibiotics (micrograms per disk): ampicillin-sulbactam (20), piperacillin-tazobactam (110), ticarcillin-clavulanate (timentin) (85), imipenem (10), meropenem (10), doripenem (10), ceftazidime (30), cefotaxime (30), cefepime (30), ceftriaxone (30), tetracycline (30), minocycline (30), doxycycline (30), streptomycin (25), spectinomycin (25), amikacin (30), kanamycin (30), gentamicin (10), neomycin (30), netilmicin (30), tobramycin (10), nalidixic acid (30), ciprofloxacin (5), levofloxacin (5), sulfamethoxazole (300), trimethoprim-sulfamethoxazole (25), and rifampin (30). The results were analyzed according to the Clinical and Laboratory Standards Institute (CLSI) recommendations for *Acinetobacter* spp. (28) and calibrated dichotomous sensitivity (CDS) (http://cdstest.net/) disk diffusion assay when a CLSI breakpoint for *Acinetobacter* spp. was not available (CDS for streptomycin, spectinomycin, kanamycin, neomycin, netilmicin, nalidixic acid, sulfamethoxazole, and rifampin).

### PCR assays

#### Identification of GC2 isolates

To identify the *A. baumannii* isolates belonging to GC2, two multiplex PCR assays were performed as described previously (29).

#### Identification of the genes conferring resistance to older and newer antibiotics carried by AbGRI1 resistance islands

**Fig 3:**
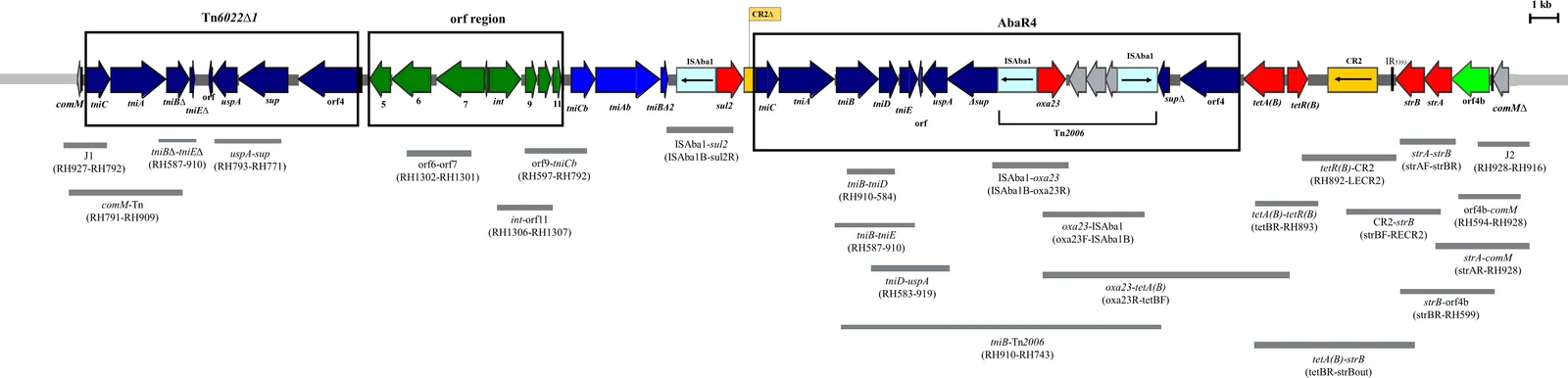
The linkage PCRs to map the backbone of AbGRI resistance islands. The central dark gray line represents the resistance island. The light gray line indicates the adjacent chromosomal sequence. Arrows indicate extent and orientation of genes and open reading frames. The resistance genes are red with the name below. Boxes represent IS and CR elements, and the internal arrows indicate the transposase or the rolling circle replicase. Vertical bars indicate IRs, and the triangles next to the genes indicate the deletion in the genes. The location of the Tn*6022*∆1, orf region, and AbaR4 is shown by black box around them. The figure is adapted with from reference 8 with permission from Dr. Blackwell.

To locate the genes conferring resistance to older antibiotics in AbGRI1, including *tetA(B), tetR(B*), *strA*, and *strB*, PCR and PCR mapping experiments, followed by sequencing confirmation were performed using the primers listed in Table S5. The *sul2* gene was detected, and whenever possible, it was linked to the AbGRI1 using the primers described previously (8). To examine whether the *oxa23* gene conferring resistance to newer antibiotics is located in AbaR4 and/or AbaR4Δ1 and also, to examine whether the AbaR4 and AbaR4Δ1 are located within AbGRI1, PCR mapping experiments were performed using the primers listed in Table S5. The linkage PCRs, which were performed to map the backbone of AbGRI1 resistance island, are shown in Fig. 3.

#### Identification of the genes conferring resistance to older antibiotics carried by AbGRI2 and AbGRI3 resistance islands

To locate the genes conferring resistance to older antibiotics in AbGRI2 (*aphA1b*, *sul1*, *aadA1*, *aacC1, bla_TEM_*) and AbGRI3 (*armA*, *aphA1b*, *sul1*, *aadA1*, *aacA4*), PCR and PCR mapping experiments, followed by sequencing confirmation were performed using the primers listed in Table S5. The PCR product of *Δatr*-ISAba24 segment was sequenced by primer walking strategy (primers are listed in Table S6).

### MLST

MLST using the Institute Pasteur (http://pubmlst.org/abaumannii/) scheme (23) was performed on selected GC2 isolates (isolates in bold, Table 1) that were representatives of each pattern (according to the type of AbGRI1, AbGRI2, and AbGRI3 resistance islands carried by isolates), followed by Oxford scheme for randomly chosen five patterns (isolates highlighted in gray, Table 1) (30).

## Data Availability

The authors declare that all data presented in this study are available within the article and its Supplementary Information Files or can be available from the corresponding author upon request. The partial sequences of the Junction 1 (J1) of AbGRI1, *tetR(B)-*CR2, AB57_1175-*tnpR*_1_, *bla_TEM-_ tnpA_1000_, tnpR_5393c_-aphA1b, tnpA_21_-*AB57_1209*,* and *armA* have been deposited in the GenBank under the following accession numbers: MW092766, OP293342, OM801571, ON240823, ON871819, OP019034, ON982224.

## AFTER EPUB

*[This article was published on 28 August 2023 with missing material in Fig. 2. The figure was corrected in the current version, posted on 12 September 2023.]*

## SUPPLEMENTAL MATERIAL

Supplemental information spectrum.05373-22-s0001.docx Tables S1 to S6.

ASM does not own the copyrights to Supplemental Material that may be linked to, or accessed through, an article. The authors have granted ASM a non-exclusive, world-wide license to publish the Supplemental Material files. Please contact the corresponding author directly for reuse.
