# Supplementary material for: Dissemination of the Acinetobacter baumannii isolates belonging to global clone 2 containing AbGRI resistance islands in a referral hospital
Source: Microbiol Spectr. 2023 Aug 28;11(5):e05373-22. doi: 10.1128/spectrum.05373-22 (PMC10581056; doi:10.1128/spectrum.05373-22)
Supplement: Supplemental information — Tables S1 to S6. [file spectrum.05373-22-s0001.docx]

**Table S1**^*^. Results of disk diffusion for GC2 *A. baumannii* isolates recovered from the hospital.

| **Isolate** | **Sm** | | **Sp** | | **Su** | | **Tc** | **Km** | | | **Nm** | | **CTX** | | **CAZ** | | **Gm** | | | **Cip** | | **AK** | | **Nx** | **Tm** | | | **Ne** | | **Ipm** | | **Mem** | | **TIM** | | **Rif** | | **SAM** | | **FEP** | | **DOR** | | **TZP** | | **CRO** | | | **MIN** | | **Dox** | | | **LVX** | | | **TS** | |  |  |  |  |
| --- | --- | --- | --- | --- | --- | --- | --- | --- | --- | --- | --- | --- | --- | --- | --- | --- | --- | --- | --- | --- | --- | --- | --- | --- | --- | --- | --- | --- | --- | --- | --- | --- | --- | --- | --- | --- | --- | --- | --- | --- | --- | --- | --- | --- | --- | --- | --- | --- | --- | --- | --- | --- | --- | --- | --- | --- | --- | --- | --- | --- | --- | --- |
| ABI001 | 6 | | 6 | | 6 | | 6 | 6 | | | 20 | | 6 | | 6 | | 6 | | 6 | | | 8 | | 6 | 6 | | | 6 | | 6 | | 6 | | 6 | | 6 | | 6 | | 6 | | 6 | | 6 | | 6 | 7 | | | | 8 | | 6 | | 6 | | | | |  |  |  |
| ABI002 | 6 | | 6 | | 6 | | 6 | 17 | | | 17 | | 6 | | 6 | | 17 | | 6 | | | 10 | | 6 | 17 | | | 17 | | 6 | | 6 | | 6 | | 6 | | 10 | | 6 | | 6 | | 8 | | 6 | 16 | | | | 6 | | 6 | | 6 | | | | |  |  |  |
| ABI004 | 6 | | 6 | | 6 | | 6 | 6 | | | 11 | | 6 | | 6 | | 9 | | 6 | | | 11 | | 6 | 6 | | | 6 | | 8 | | 6 | | 6 | | 6 | | 8 | | 6 | | 6 | | 8 | | 6 | 8 | | | | 8 | | 6 | | 6 | | | | |  |  |  |
| ABI005 | 6 | | 6 | | 10 | | 6 | 6 | | | 18 | | 6 | | 6 | | 6 | | 6 | | | 6 | | 6 | 6 | | | 6 | | 6 | | 6 | | 6 | | 8 | | 10 | | 6 | | 6 | | 7 | | 6 | 7 | | | | 6 | | 7 | | 6 | | | | |  |  |  |
| ABI007 | 6 | | 6 | | 6 | | 10 | 6 | | | 6 | | 6 | | 6 | | 25 | | 6 | | | 20 | | 6 | 19 | | | 18 | | 6 | | 7 | | 6 | | 12 | | 9 | | 7 | | 6 | | 8 | | 6 | 23 | | | | 23 | | 9 | | 20 | | | | |  |  |  |
| ABI014 | 6 | | 6 | | 6 | | 6 | 6 | | | 6 | | 6 | | 6 | | 6 | | 6 | | | 9 | | 6 | 6 | | | 13 | | 6 | | 6 | | 6 | | 12 | | 14 | | 8 | | 6 | | 8 | | 6 | 12 | | | | 10 | | 10 | | 6 | | | | |  |  |  |
| ABI020 | 6 | | 6 | | 6 | | 6 | 6 | | | 20 | | 6 | | 6 | | 6 | | 6 | | | 6 | | 6 | 6 | | | 13 | | 6 | | 6 | | 6 | | 6 | | 8 | | 6 | | 6 | | 6 | | 6 | 12 | | | | 8 | | 6 | | 6 | | | | |  |  |  |
| ABI024 | 11 | | 6 | | 10 | | 6 | 18 | | | 18 | | 6 | | 6 | | 17 | | 6 | | | 6 | | 6 | 19 | | | 17 | | 6 | | 6 | | 6 | | 12 | | 10 | | 6 | | 6 | | 8 | | 6 | 12 | | | | 8 | | 8 | | 6 | | | | |  |  |  |
| ABI026 | 10 | | 6 | | 6 | | 10 | 6 | | | 6 | | 6 | | 6 | | 6 | | 6 | | | 6 | | 6 | 6 | | | 6 | | 6 | | 6 | | 6 | | 11 | | 10 | | 6 | | 6 | | 7 | | 6 | 15 | | | | 7 | | 6 | | 6 | | | | |  |  |  |
| ABI028 | 6 | | 6 | | 6 | | 6 | 6 | | | 6 | | 6 | | 6 | | 6 | | 6 | | | 6 | | 6 | 6 | | | 6 | | 6 | | 6 | | 6 | | 11 | | 8 | | 10 | | 6 | | 6 | | 6 | 12 | | | | 8 | | 9 | | 6 | | | | |  |  |  |
| ABI034 | 6 | | 6 | | 6 | | 6 | 6 | | | 6 | | 6 | | 6 | | 6 | | 6 | | | 6 | | 6 | 6 | | | 6 | | 6 | | 6 | | 6 | | 12 | | 7 | | 6 | | 6 | | 7 | | 6 | 8 | | | | 8 | | 9 | | 6 | | | | |  |  |  |
| ABI045 | 11 | | 6 | | 6 | | 6 | 6 | | | 13 | | 6 | | 6 | | 6 | | 6 | | | 6 | | 6 | 6 | | | 21 | | 6 | | 6 | | 6 | | 12 | | 8 | | 6 | | 6 | | 7 | | 6 | 12 | | | | 7 | | 6 | | 6 | | | | |  |  |  |
| ABI046 | 11 | | 7 | | 6 | | 9 | 6 | | | 6 | | 6 | | 6 | | 6 | | 6 | | | 6 | | 6 | 6 | | | 6 | | 6 | | 6 | | 6 | | 10 | | 8 | | 6 | | 6 | | 9 | | 6 | 12 | | | | 7 | | 6 | | 6 | | | | |  |  |  |
| ABI052 | 6 | | 6 | | 6 | | 10 | 17 | | | 17 | | 6 | | 6 | | 15 | | 6 | | | 6 | | 6 | 17 | | | 17 | | 10 | | 6 | | 6 | | 13 | | 7 | | 7 | | 6 | | 12 | | 6 | 16 | | | | 6 | | 7 | | 6 | | | | |  |  |  |
| ABI076 | 11 | | 6 | | 6 | | 10 | 6 | | | 10 | | 6 | | 6 | | 6 | | 6 | | | 10 | | 6 | 6 | | | 6 | | 6 | | 6 | | 6 | | 11 | | 6 | | 7 | | 6 | | 7 | | 6 | 16 | | | | 6 | | 6 | | 6 | | | | |  |  |  |
| ABI143 | 11 | | 6 | | 6 | | 10 | 6 | | | 10 | | 6 | | 6 | | 6 | | 6 | | | 6 | | 6 | 6 | | | 6 | | 6 | | 6 | | 6 | | 12 | | 6 | | 7 | | 6 | | 7 | | 6 | 11 | | | | 6 | | 10 | | 6 | | | | |  |  |  |
| ABI151 | 6 | | 6 | | 6 | | 6 | 6 | | | 17 | | 6 | | 6 | | 10 | | 6 | | | 6 | | 6 | 6 | | | 6 | | 6 | | 6 | | 6 | | 7 | | 8 | | 6 | | 6 | | 7 | | 6 | 20 | | | | 13 | | 8 | | 6 | | | | |  |  |  |
| ABI154 | 6 | | 6 | | 6 | | 7 | 20 | | | 15 | | 6 | | 6 | | 15 | | 6 | | | 20 | | 6 | 17 | | | 17 | | 6 | | 6 | | 6 | | 8 | | 7 | | 6 | | 6 | | 7 | | 6 | 17 | | | | 8 | | 8 | | 6 | | | | |  |  |  |
| ABI157 | 6 | | 6 | | 6 | | 8 | 17 | | | 18 | | 6 | | 6 | | 16 | | 6 | | | 6 | | 6 | 18 | | | 18 | | 6 | | 6 | | 6 | | 7 | | 10 | | 6 | | 6 | | 10 | | 6 | 23 | | | | 15 | | 8 | | 6 | | | | |  |  |  |
| ABI159 | 6 | | 6 | | 6 | | 8 | 6 | | | 6 | | 6 | | 6 | | 12 | | 6 | | | 6 | | 6 | 6 | | | 6 | | 6 | | 6 | | 6 | | 9 | | 6 | | 6 | | 6 | | 6 | | 6 | 11 | | | | 6 | | 8 | | 6 | | | | |  |  |  |
| ABI163 | 6 | | 6 | | 6 | | 6 | 18 | | | 6 | | 6 | | 6 | | 15 | | 6 | | | 14 | | 6 | 17 | | | 18 | | 6 | | 6 | | 6 | | 10 | | 10 | | 8 | | 6 | | 10 | | 6 | 17 | | | | 6 | | 9 | | 6 | | | | |  |  |  |
| ABI166 | 13 | | 10 | | 6 | | 10 | 6 | | | 6 | | 6 | | 6 | | 6 | | 6 | | | 6 | | 6 | 6 | | | 6 | | 6 | | 6 | | 6 | | 6 | | 10 | | 10 | | 6 | | 10 | | 6 | 12 | | | | 12 | | 6 | | 6 | | | | |  |  |  |
| ABI167 | 6 | | 6 | | 6 | | 6 | 17 | | | 10 | | 6 | | 6 | | 16 | | 6 | | | 18 | | 6 | 17 | | | 17 | | 6 | | 6 | | 6 | | 11 | | 11 | | 6 | | 6 | | 10 | | 6 | 23 | | | | 14 | | 8 | | 6 | | | | |  |  |  |
| ABI169 | 6 | | 6 | | 6 | | 6 | 6 | | | 6 | | 6 | | 6 | | 12 | | 6 | | | 6 | | 6 | 6 | | | 6 | | 6 | | 6 | | 6 | | 10 | | 10 | | 9 | | 6 | | 10 | | 6 | 18 | | | | 12 | | 10 | | 6 | | | | |  |  |  |
| ABI171 | 13 | | 10 | | 6 | | 11 | 6 | | | 20 | | 6 | | 6 | | 6 | | 6 | | | 6 | | 6 | 6 | | | 6 | | 6 | | 6 | | 6 | | 9 | | 7 | | 7 | | 6 | | 6 | | 6 | 21 | | | | 14 | | 7 | | 6 | | | | |  |  |  |
| ABI179 | 12 | | 7 | | 6 | | 9 | 6 | | | 17 | | 6 | | 6 | | 6 | | 6 | | | 6 | | 6 | 6 | | | 6 | | 6 | | 6 | | 6 | | 10 | | 8 | | 7 | | 6 | | 7 | | 6 | 26 | | | | 17 | | 6 | | 6 | | | | |  |  |  |
| ABI181 | 6 | | 6 | | 6 | | 8 | 17 | | | 15 | | 6 | | 6 | | 16 | | 6 | | | 19 | | 6 | 18 | | | 17 | | 6 | | 6 | | 6 | | 6 | | 8 | | 6 | | 6 | | 8 | | 6 | 18 | | | | 7 | | 6 | | 6 | | | | |  |  |  |
| ABI184 | 11 | | 8 | | 6 | | 11 | 6 | | | 6 | | 6 | | 6 | | 6 | | 6 | | | 6 | | 6 | 18 | | | 17 | | 6 | | 6 | | 6 | | 11 | | 9 | | 7 | | 6 | | 10 | | 6 | 9 | | | | 6 | | 6 | | 10 | | | | |  |  |  |
| ABI185 | 12 | | 7 | | 6 | | 11 | 6 | | | 17 | | 6 | | 6 | | 6 | | 6 | | | 6 | | 6 | 6 | | | 6 | | 6 | | 6 | | 6 | | 10 | | 6 | | 6 | | 6 | | 8 | | 6 | 21 | | | | 14 | | 6 | | 6 | | | | |  |  |  |
| ABI188 | 6 | | 6 | | 6 | | 10 | 6 | | | 6 | | 6 | | 6 | | 6 | | 6 | | | 6 | | 6 | 18 | | | 18 | | 6 | | 6 | | 6 | | 11 | | 10 | | 7 | | 6 | | 10 | | 6 | 8 | | | | 6 | | 6 | | 6 | | | | |  |  |  |
| ABI189 | 6 | | 6 | | 6 | | 11 | 6 | | | 7 | | 6 | | 6 | | 11 | | 6 | | | 13 | | 6 | 6 | | | 8 | | 6 | | 6 | | 6 | | 11 | | 12 | | 6 | | 6 | | 6 | | 6 | 16 | | | | 6 | | 6 | | 6 | | | | |  |  |  |
| ABI190 | 13 | | 6 | | 6 | | 10 | 6 | | | 17 | | 6 | | 6 | | 6 | | 6 | | | 6 | | 6 | 6 | | | 6 | | 6 | | 6 | | 6 | | 12 | | 8 | | 8 | | 6 | | 9 | | 6 | 21 | | | | 15 | | 6 | | 6 | | | | |  |  |  |
| ABI191 | 13 | | 6 | | 6 | | 11 | 6 | | | 6 | | 6 | | 6 | | 6 | | 6 | | | 6 | | 6 | 6 | | | 6 | | 6 | | 6 | | 6 | | 15 | | 10 | | 10 | | 6 | | 6 | | 6 | 17 | | | | 11 | | 6 | | 6 | | | | |  |  |  |
| ABI192 | 13 | | 8 | | 6 | | 6 | 6 | | | 6 | | 6 | | 6 | | 6 | | 6 | | | 6 | | 6 | 6 | | | 6 | | 6 | | 6 | | 6 | | 10 | | 8 | | 8 | | 6 | | 6 | | 6 | 16 | | | | 11 | | 6 | | 6 | | | | |  |  |  |
| ABI193 | 6 | | 6 | | 6 | | 6 | 6 | | | 6 | | 6 | | 6 | | 6 | | 6 | | | 6 | | 6 | 6 | | | 6 | | 6 | | 6 | | 6 | | 10 | | 8 | | 8 | | 6 | | 6 | | 6 | 10 | | | | 6 | | 8 | | 6 | | | | |  |  |  |
| ABI194 | 13 | | 6 | | 6 | | 6 | 6 | | | 6 | | 6 | | 6 | | 6 | | 6 | | | 6 | | 6 | 6 | | | 6 | | 6 | | 6 | | 6 | | 10 | | 8 | | 8 | | 6 | | 6 | | 6 | 21 | | | | 20 | | 6 | | 6 | | | | |  |  |  |
| ABI195 | 6 | | 6 | | 6 | | 10 | 6 | | | 6 | | 6 | | 6 | | 11 | | 6 | | | 6 | | 6 | 17 | | | 17 | | 6 | | 6 | | 6 | | 10 | | 8 | | 8 | | 6 | | 6 | | 6 | 18 | | | | 20 | | 10 | | 6 | | | | |  |  |  |
| ABI196 | 6 | | 6 | | 25 | | 6 | 17 | | | 8 | | 6 | | 6 | | 15 | | 6 | | | 6 | | 6 | 18 | | | 17 | | 6 | | 6 | | 6 | | 6 | | 8 | | 6 | | 6 | | 6 | | 6 | 10 | | | | 6 | | 6 | | 6 | | | | |  |  |  |
| ABI198 | 12 | | 6 | | 6 | | 12 | 6 | | | 6 | | 6 | | 6 | | 6 | | 6 | | | 6 | | 6 | 6 | | | 6 | | 6 | | 6 | | 6 | | 12 | | 7 | | 7 | | 6 | | 10 | | 6 | 23 | | | | 20 | | 10 | | 6 | | | | |  |  |  |
| ABI199 | 6 | | 6 | | 6 | | 17 | 6 | | | 6 | | 6 | | 6 | | 6 | | 6 | | | 6 | | 6 | 6 | | | 6 | | 6 | | 6 | | 6 | | 10 | | 8 | | 6 | | 8 | | 8 | | 6 | 18 | | | | 16 | | 10 | | 6 | | | | |  |  |  |
| ABI201 | 12 | | 9 | | 27 | | 10 | 6 | | | 6 | | 6 | | 6 | | 12 | | 6 | | | 6 | | 6 | 6 | | | 6 | | 6 | | 6 | | 6 | | 12 | | 15 | | 6 | | 6 | | 6 | | 6 | 12 | | | | 6 | | 6 | | 6 | | | | |  |  |  |
| ABI203 | 9 | | 6 | | 25 | | 10 | 6 | | | 6 | | 6 | | 6 | | 12 | | 6 | | | 6 | | 6 | 6 | | | 10 | | 6 | | 6 | | 6 | | 12 | | 15 | | 8 | | 6 | | 6 | | 6 | 10 | | | | 6 | | 8 | | 6 | | | | |  |  |  |
| ABI204 | 6 | | 6 | | 6 | | 17 | 6 | | | 6 | | 6 | | 6 | | 12 | | 6 | | | 6 | | 6 | 6 | | | 12 | | 6 | | 6 | | 6 | | 10 | | 8 | | 8 | | 6 | | 6 | | 6 | 25 | | | | 23 | | 6 | | 6 | | | | |  |  |  |
| ABI205 | 6 | | 6 | | 6 | | 6 | 6 | | | 6 | | 6 | | 6 | | 12 | | 6 | | | 6 | | 6 | 6 | | | 6 | | 6 | | 6 | | 6 | | 10 | | 8 | | 6 | | 6 | | 6 | | 6 | 23 | | | | 23 | | 6 | | 6 | | | | |  |  |  |
| ABI206 | 6 | | 6 | | 27 | | 6 | 6 | | | 6 | | 6 | | 6 | | 6 | | 6 | | | 6 | | 6 | 6 | | | 6 | | 6 | | 6 | | 6 | | 12 | | 15 | | 8 | | 6 | | 6 | | 6 | 7 | | | | 6 | | 6 | | 13 | | | | |  |  |  |
| ABI207 | 13 | | 12 | | 27 | | 8 | 6 | | | 6 | | 6 | | 6 | | 6 | | 6 | | | 6 | | 6 | 6 | | | 6 | | 6 | | 6 | | 6 | | 9 | | 8 | | 7 | | 6 | | 6 | | 6 | 12 | | | | 6 | | 6 | | 15 | | | | |  |  |  |
| ABI215 | 13 | | 12 | | 27 | | 8 | 6 | | | 6 | | 6 | | 6 | | 6 | | 6 | | | 14 | | 6 | 6 | | | 6 | | 6 | | 6 | | 6 | | 12 | | 15 | | 8 | | 6 | | 6 | | 6 | 11 | | | | 6 | | 11 | | 15 | | | | |  |  |  |
| ABI216 | 6 | | 6 | | 6 | | 7 | 6 | | | 8 | | 6 | | 6 | | 12 | | 6 | | | 6 | | 6 | 6 | | | 6 | | 6 | | 6 | | 6 | | 10 | | 8 | | 6 | | 6 | | 6 | | 6 | 11 | | | | 6 | | 7 | | 6 | | | | |  |  |  |
| ABI219 | 9 | | 6 | | 6 | | 6 | 6 | | | 6 | | 6 | | 6 | | 6 | | 6 | | | 6 | | 6 | 6 | | | 6 | | 6 | | 6 | | 6 | | 10 | | 9 | | 6 | | 6 | | 9 | | 6 | 6 | | | | 6 | | 6 | | 6 | | | | |  |  |  |
| ABI220 | 14 | | 14 | | 6 | | 13 | 16 | | | 16 | | 6 | | 6 | | 15 | | 6 | | | 6 | | 6 | 17 | | | 18 | | 6 | | 6 | | 6 | | 9 | | 8 | | 7 | | 6 | | 6 | | 6 | 21 | | | | 15 | | 6 | | 6 | | | | |  |  |  |
| ABI221 | 6 | | 6 | | 6 | | 6 | 6 | | | 16 | | 6 | | 6 | | 6 | | 6 | | | 6 | | 6 | 6 | | | 6 | | 6 | | 6 | | 6 | | 9 | | 8 | | 7 | | 6 | | 6 | | 6 | 16 | | | | 6 | | 6 | | 6 | | | | |  |  |  |
| ABI222 | 7 | | 6 | | 6 | | 8 | 6 | | | 6 | | 6 | | 6 | | 6 | | 6 | | | 6 | | 6 | 6 | | | 6 | | 6 | | 6 | | 6 | | 9 | | 7 | | 6 | | 6 | | 6 | | 6 | 16 | | | | 15 | | 8 | | 6 | | | | |  |  |  |
| ABI223 | 6 | | 6 | | 10 | | 12 | 6 | | | 16 | | 6 | | 6 | | 10 | | 6 | | | 6 | | 6 | 12 | | | 6 | | 6 | | 6 | | 6 | | 10 | | 11 | | 6 | | 6 | | 6 | | 6 | 18 | | | | 17 | | 6 | | 6 | | | | |  |  |  |
| ABI227 | 6 | | 6 | | 6 | | 10 | 6 | | | 6 | | 6 | | 6 | | 6 | | 6 | | | 6 | | 6 | 6 | | | 6 | | 6 | | 6 | | 6 | | 13 | | 15 | | 7 | | 7 | | 6 | | 6 | 8 | | | | 6 | | 7 | | 6 | | | | |  |  |  |
| ABI229 | 6 | | 6 | | 12 | | 12 | 6 | | | 6 | | 6 | | 6 | | 17 | | 6 | | | 19 | | 6 | 6 | | | 17 | | 6 | | 6 | | 6 | | 12 | | 7 | | 6 | | 6 | | 6 | | 6 | 11 | | | | 17 | | 6 | | 6 | | | | |  |  |  |
| ABI230 | 7 | | 6 | | 6 | | 13 | 6 | | | 17 | | 6 | | 6 | | 12 | | 6 | | | 6 | | 6 | 12 | | | 6 | | 6 | | 6 | | 6 | | 10 | | 8 | | 7 | | 6 | | 6 | | 6 | 20 | | | | 17 | | 7 | | 6 | | | | |  |  |  |
| ABI231 | 11 | | 6 | | 6 | | 10 | 6 | | | 17 | | 6 | | 6 | | 12 | | 6 | | | 6 | | 6 | 6 | | | 6 | | 6 | | 6 | | 6 | | 10 | | 8 | | 7 | | 6 | | 6 | | 6 | 12 | | | | 10 | | 6 | | 6 | | | | |  |  |  |
| ABI233 | 10 | | 6 | | 25 | | 10 | 6 | | | 16 | | 6 | | 6 | | 12 | | 6 | | | 6 | | 6 | 6 | | | 6 | | 6 | | 6 | | 6 | | 13 | | 15 | | 7 | | 6 | | 6 | | 6 | 9 | | | | 10 | | 7 | | 6 | | | | |  |  |  |
| ABI239 | 11 | | 6 | | 6 | | 8 | 6 | | | 6 | | 6 | | 6 | | 6 | | 6 | | | 6 | | 6 | 6 | | | 6 | | 6 | | 6 | | 6 | | 13 | | 9 | | 8 | | 6 | | 6 | | 6 | 11 | | | | 6 | | 6 | | 17 | | | | |  |  |  |
| ABI242 | 11 | | 6 | | 6 | | 13 | 6 | | | 16 | | 6 | | 6 | | 12 | | 6 | | | 6 | | 6 | 6 | | | 6 | | 6 | | 6 | | 6 | | 10 | | 9 | | 8 | | 6 | | 6 | | 6 | 11 | | | | 18 | | 6 | | 6 | | | | |  |  |  |
| ABI243 | 6 | | 6 | | 6 | | 6 | 6 | | | 16 | | 6 | | 6 | | 6 | | 6 | | | 15 | | 6 | 6 | | | 6 | | 6 | | 6 | | 6 | | 10 | | 6 | | 6 | | 6 | | 6 | | 6 | 16 | | | | 6 | | 11 | | 6 | | | | |  |  |  |
| ABI248 | 6 | | 6 | | 6 | | 6 | 6 | | | 20 | | 6 | | 6 | | 11 | | 6 | | | 6 | | 6 | 6 | | | 6 | | 6 | | 6 | | 6 | | 12 | | 15 | | 8 | | 6 | | 6 | | 6 | 10 | | | | 6 | | 8 | | 13 | | | | |  |  |  |
| ABI249 | 11 | | 6 | | 6 | | 7 | 6 | | | 8 | | 6 | | 6 | | 6 | | 6 | | | 6 | | 6 | 6 | | | 6 | | 6 | | 6 | | 6 | | 10 | | 9 | | 8 | | 6 | | 6 | | 6 | 16 | | | | 13 | | 6 | | 6 | | | | |  |  |  |
| ABI250 | 6 | | 6 | | 6 | | 6 | 6 | | | 16 | | 6 | | 6 | | 12 | | 6 | | | 6 | | 6 | 6 | | | 6 | | 6 | | 6 | | 6 | | 10 | | 10 | | 8 | | 6 | | 6 | | 6 | 12 | | | | 6 | | 8 | | 6 | | | | |  |  |  |
| ABI251 | 6 | | 7 | | 22 | | 6 | 6 | | | 6 | | 6 | | 6 | | 12 | | 6 | | | 6 | | 6 | 6 | | | 6 | | 6 | | 6 | | 6 | | 10 | | 12 | | 9 | | 6 | | 6 | | 6 | 10 | | | | 6 | | 6 | | 12 | | | | |  |  |  |
| ABI252 | 11 | | 6 | | 6 | | 10 | 6 | | | 6 | | 6 | | 6 | | 6 | | 6 | | | 6 | | 6 | 6 | | | 6 | | 6 | | 6 | | 6 | | 10 | | 9 | | 8 | | 6 | | 9 | | 6 | 16 | | | | 7 | | 6 | | 6 | | | | |  |  |  |
| ABI254 | 11 | | 6 | | 6 | | 13 | 6 | | | 6 | | 6 | | 6 | | 12 | | 6 | | | 6 | | 6 | 6 | | | 6 | | 6 | | 6 | | 6 | | 10 | | 9 | | 10 | | 6 | | 8 | | 6 | 21 | | | | 20 | | 11 | | 6 | | | | |  |  |  |
| ABI255 | 6 | | 6 | | 6 | | 6 | 6 | | | 17 | | 6 | | 6 | | 12 | | 6 | | | 15 | | 6 | 12 | | | 6 | | 6 | | 6 | | 6 | | 12 | | 10 | | 8 | | 6 | | 9 | | 6 | 12 | | | | 6 | | 7 | | 6 | | | | |  |  |  |
| ABI256 | 6 | | 7 | | 6 | | 6 | 6 | | | 6 | | 6 | | 6 | | 12 | | 6 | | | 6 | | 6 | 6 | | | 6 | | 6 | | 6 | | 6 | | 10 | | 13 | | 8 | | 6 | | 6 | | 6 | 10 | | | | 6 | | 8 | | 7 | | | | |  |  |  |
| ABI257 | 6 | | 6 | | 6 | | 6 | 6 | | | 17 | | 6 | | 6 | | 12 | | 6 | | | 15 | | 6 | 6 | | | 13 | | 6 | | 6 | | 6 | | 6 | | 10 | | 8 | | 6 | | 6 | | 6 | 16 | | | | 6 | | 9 | | 6 | | | | |  |  |  |
| ABI258 | 6 | | 6 | | 6 | | 6 | 6 | | | 16 | | 6 | | 6 | | 12 | | 6 | | | 12 | | 6 | 6 | | | 6 | | 6 | | 6 | | 6 | | 6 | | 10 | | 6 | | 6 | | 9 | | 6 | 16 | | | | 6 | | 9 | | 6 | | | | |  |  |  |
| ABI259 | 6 | | 6 | | 6 | | 17 | 6 | | | 6 | | 6 | | 6 | | 6 | | 6 | | | 13 | | 6 | 6 | | | 6 | | 6 | | 6 | | 6 | | 10 | | 11 | | 9 | | 6 | | 9 | | 6 | 18 | | | | 6 | | 8 | | 6 | | | | |  |  |  |
| ABI260 | 6 | | 6 | | 6 | | 6 | 6 | | | 20 | | 6 | | 6 | | 6 | | 6 | | | 6 | | 6 | 6 | | | 6 | | 6 | | 6 | | 6 | | 13 | | 15 | | 6 | | 6 | | 10 | | 6 | 10 | | | | 6 | | 6 | | 6 | | | | |  |  |  |
| ABI261 | 6 | | 6 | | 6 | | 6 | 6 | | | 20 | | 6 | | 6 | | 6 | | 6 | | | 14 | | 6 | 6 | | | 6 | | 6 | | 6 | | 6 | | 10 | | 11 | | 6 | | 6 | | 10 | | 6 | 16 | | | | 6 | | 8 | | 10 | | | | |  |  |  |
| ABI262 | 6 | | 6 | | 6 | | 6 | 6 | | | 20 | | 6 | | 6 | | 12 | | 6 | | | 6 | | 6 | 6 | | | 6 | | 6 | | 6 | | 6 | | 10 | | 11 | | 6 | | 6 | | 9 | | 6 | 7 | | | | 6 | | 6 | | 10 | | | | |  |  |  |
| ABI263 | 6 | | 6 | | 6 | | 6 | 6 | | | 20 | | 6 | | 6 | | 6 | | 6 | | | 6 | | 6 | 6 | | | 6 | | 6 | | 6 | | 6 | | 10 | | 13 | | 6 | | 6 | | 10 | | 6 | 16 | | | | 6 | | 8 | | 6 | | | | |  |  |  |
| ABI265 | 11 | | 6 | | 6 | | 6 | 6 | | | 21 | | 6 | | 6 | | 12 | | 6 | | | 14 | | 6 | 6 | | | 6 | | 6 | | 6 | | 6 | | 12 | | 15 | | 6 | | 6 | | 10 | | 6 | 9 | | | | 6 | | 6 | | 6 | | | | |  |  |  |
| ABI266 | 11 | | 6 | | 6 | | 10 | 6 | | | 15 | | 6 | | 6 | | 6 | | 6 | | | 12 | | 6 | 6 | | | 6 | | 6 | | 6 | | 6 | | 14 | | 15 | | 6 | | 6 | | 10 | | 6 | 10 | | | | 6 | | 8 | | 6 | | | | |  |  |  |
| ABI268 | 6 | | 6 | | 6 | | 6 | 6 | | | 16 | | 6 | | 6 | | 6 | | 6 | | | 13 | | 6 | 6 | | | 6 | | 6 | | 6 | | 6 | | 10 | | 11 | | 6 | | 6 | | 10 | | 6 | 16 | | | | 6 | | 7 | | 10 | | | | |  |  |  |
| ABI269 | 6 | | 6 | | 6 | | 6 | 6 | | | 6 | | 6 | | 6 | | 6 | | 6 | | | 14 | | 6 | 19 | | | 17 | | 6 | | 6 | | 6 | | 10 | | 10 | | 6 | | 6 | | 9 | | 6 | 10 | | | | 6 | | 8 | | 6 | | | | |  |  |  |
| ABI270 | 6 | | 6 | | 14 | | 6 | 6 | | | 17 | | 6 | | 6 | | 6 | | 6 | | | 6 | | 6 | 6 | | | 6 | | 6 | | 6 | | 6 | | 10 | | 11 | | 6 | | 6 | | 10 | | 6 | 16 | | | | 6 | | 6 | | 6 | | | | |  |  |  |
| ABI271 | 6 | | 6 | | 6 | | 6 | 6 | | | 17 | | 6 | | 6 | | 6 | | 6 | | | 6 | | 6 | 6 | | | 6 | | 8 | | 6 | | 6 | | 10 | | 12 | | 6 | | 6 | | 6 | | 6 | 14 | | | | 6 | | 6 | | 6 | | | | |  |  |  |
| ABI272 | 6 | | 6 | | 23 | | 6 | 6 | | | 17 | | 6 | | 6 | | 6 | | 6 | | | 6 | | 6 | 6 | | | 6 | | 6 | | 6 | | 6 | | 10 | | 15 | | 6 | | 6 | | 10 | | 6 | 11 | | | | 6 | | 6 | | 10 | | | | |  |  |  |
| ABI273 | 6 | | 6 | | 6 | | 6 | 6 | | | 13 | | 6 | | 6 | | 11 | | 6 | | | 13 | | 6 | 6 | | | 6 | | 6 | | 6 | | 6 | | 12 | | 10 | | 6 | | 6 | | 9 | | 6 | 12 | | | | 6 | | 6 | | 6 | | | | |  |  |  |
| ABI275 | 6 | | 6 | | 6 | | 6 | 16 | | | 13 | | 6 | | 6 | | 16 | | 6 | | | 6 | | 6 | 17 | | | 18 | | 6 | | 6 | | 6 | | 6 | | 10 | | 6 | | 6 | | 10 | | 6 | 14 | | | | 11 | | 6 | | 6 | | | | |  |  |  |
| ABI277 | 6 | | 6 | | 6 | | 6 | 6 | | | 20 | | 6 | | 6 | | 6 | | 6 | | | 6 | | 6 | 6 | | | 6 | | 8 | | 6 | | 6 | | 10 | | 13 | | 6 | | 6 | | 10 | | 6 | 15 | | | | 11 | | 6 | | 10 | | | | |  |  |  |
| ABI281 | 6 | | 6 | | 6 | | 6 | 6 | | | 20 | | 6 | | 6 | | 6 | | 6 | | | 6 | | 6 | 6 | | | 6 | | 6 | | 6 | | 6 | | 10 | | 12 | | 6 | | 6 | | 10 | | 6 | 16 | | | | 11 | | 7 | | 10 | | | | |  |  |  |
| ABI283 | 6 | | 6 | | 6 | | 10 | 6 | | | 20 | | 6 | | 6 | | 6 | | 6 | | | 6 | | 6 | 6 | | | 6 | | 6 | | 6 | | 6 | | 10 | | 6 | | 6 | | 6 | | 6 | | 6 | 13 | | | | 6 | | 6 | | 12 | | | | |  |  |  |
| ABI284 | 6 | | 6 | | 6 | | 17 | 6 | | | 20 | | 6 | | 6 | | 6 | | 6 | | | 6 | | 6 | 6 | | | 6 | | 6 | | 6 | | 6 | | 10 | | 8 | | 6 | | 6 | | 6 | | 6 | 23 | | | | 20 | | 6 | | 6 | | | | |  |  |  |
| ABI286 | 6 | | 6 | | 6 | | 6 | 6 | | | 20 | | 6 | | 6 | | 11 | | 6 | | | 6 | | 6 | 6 | | | 6 | | 6 | | 6 | | 6 | | 11 | | 15 | | 9 | | 6 | | 6 | | 6 | 11 | | | | 6 | | 12 | | 6 | | | | |  |  |  |
|  |  |  | |  | |  | | |  |  | |  | |  | |  | |  | | |  | |  | | |  |  | |  | |  | |  | |  | |  | |  | |  | |  | |  | | |  | |  | |  | | | |  | |  | | |  |  |

* Sm: Streptomycin, Sp: Spectinomycin, Su: Sulfamethoxazole, Tc: Tetracycline, Km: Kanamycin, Nm: Neomycin, CTX: Cefotaxime, CAZ: Ceftazidime, Gm: Gentamicin, Cip: Ciprofloxacin, AK: Amikacin, Nx: Nalidixic Acid, Tm: Tobramycin, Ne: Netilmicin, Ipm: Imipenem, Mem: Meropenem, TIM: Timentin (Ticarcillin-clavulanic acid), Rif: Rifampicin, SAM: Ampicillin-sulbactam, FEP: Cefepime, DOR: Doripenem, TZP: Piperacillin/Tazobactam, CRO: Ceftriaxone, MIN: Minocycline, DOX: Doxycycline, LVX: Levofloxacin, TS: Trimethoprim-sulfamethoxazole. Inhibition zone diameters highlighted white, light gray and dark gray indicate susceptibility, intermediate susceptibility, and resistance, respectively.

**Table S2**. Characteristics of the GC2 isolates containing AbGRI1 resistance island.

| Isolate | Isolation date | Ward | Source | *comM* | J1 | J2 | orf4b-*comM* | *tniBΔ-tniEΔ* | *tniB-tniE* | *tniB-tniD* | *tniD-uspA* | *comM* -Tn | *sul2* | ISAba1- *sul2* | *strA* | *strB* | *strA-strB* | *strA-comM* | *strB -* orf4b | CR2 | **CR2*-* *strB*** | *tetA(B)* | *tetR(B)* | ***tetA(B)- tetR(B)*** | *tetA(B)- strB* | *tetR(B)-* CR2 | orf6- orf7 | *int*- orf11 | orf9- *tniCb* | *oxa23* | ISAba1- *oxa23* | *oxa23-* ISAba1 | *tniB-*Tn*2006* | *oxa23-* *tetA(B)* |
| --- | --- | --- | --- | --- | --- | --- | --- | --- | --- | --- | --- | --- | --- | --- | --- | --- | --- | --- | --- | --- | --- | --- | --- | --- | --- | --- | --- | --- | --- | --- | --- | --- | --- | --- |
| ABI001 | November 2011 | ICU 10 | Blood | - | + | + | + | + | + | + | + | + | + | + | + | + | + | + | + | + | + | + | + | + | + | + | + | + | + | + | + | + | + | - |
| ABI002 | November 2011 | ICU 10 | Blood | - | + | + | + | + | - | - | - | + | + | + | + | + | + | + | + | + | + | + | + | + | + | + | + | + | + | + | + | + | + | - |
| ABI004 | November 2011 | Ward 21 | Catheter | - | + | + | + | + | + | + | + | + | + | + | + | + | + | + | + | + | + | + | + | + | + | + | + | + | + | + | + | + | + | + |
| ABI005 | November 2011 | Ward 13 | Ascites | - | + | + | + | + | + | + | + | + | + | + | + | + | + | + | + | + | + | + | + | + | + | + | + | + | + | + | + | + | + | + |
| ABI007 | November 2011 | ICU 10 | Blood | - | + | + | + | + | - | - | - | + | + | + | + | + | + | + | + | + | + | + | + | + | + | + | + | + | + | + | + | + | + | + |
| ABI014 | December 2011 | NR | Trachea | - | + | + | + | + | + | + | + | + | + | + | + | + | + | + | + | + | + | + | + | + | + | + | + | + | + | + | + | + | + | + |
| ABI020 | December 2011 | ICU 10 | Trachea | - | + | + | + | + | + | + | + | + | + | + | + | + | + | + | + | + | + | + | + | + | + | + | + | + | + | + | + | + | + | + |
| ABI024 | January 2012 | Ward 5 | Trachea | - | + | + | + | + | - | - | - | + | + | + | + | + | + | + | + | + | + | + | + | + | + | + | + | + | + | + | + | + | + | - |
| ABI026 | January 2012 | Ward 5 | NR | - | + | + | + | + | + | + | + | + | + | + | + | + | + | + | + | + | + | + | + | + | + | + | + | + | + | + | + | + | + | + |
| ABI028 | April 2012 | NR | Trachea | - | + | + | + | + | + | + | + | + | + | + | + | + | + | + | + | + | + | + | + | + | + | + | + | + | + | + | + | + | - | ND |
| ABI034 | January 2012 | ICU 10 | Wound | - | + | + | + | + | + | + | + | + | + | + | + | + | + | + | + | + | + | + | + | + | + | + | + | + | + | + | + | + | + | - |
| ABI045 | May 2012 | NR | Wound | - | + | + | + | - | + | + | + | + | + | + | + | + | + | + | + | + | + | + | + | + | + | + | - | - | - | + | + | + | + | - |
| ABI046 | May 2012 | NR | Wound | - | + | + | + | + | + | + | + | + | + | + | + | + | + | + | + | + | + | + | + | + | + | + | + | + | + | + | + | + | + | + |
| ABI052 | June 2012 | NR | NR | - | + | + | + | + | - | - | - | + | + | + | + | + | + | + | + | + | + | + | + | + | + | + | + | + | + | + | + | + | + | + |
| ABI076 | September 2012 | NR | NR | - | + | + | + | + | + | + | + | + | + | + | + | + | + | + | + | + | + | + | + | + | + | + | + | + | + | + | + | + | + | - |
| ABI143 | December 2018 | ICU 3 | Trachea | - | + | + | + | + | + | + | + | + | + | + | + | + | + | + | + | + | + | + | + | + | + | + | + | + | + | + | + | + | + | - |
| ABI154 | December 2018 | ICU 2 | Discharges | - | + | + | + | + | - | - | - | + | + | + | + | + | + | + | + | + | + | + | + | + | + | + | + | + | + | + | + | + | - | ND |
| ABI157 | December 2018 | ICU 10 | Trachea | - | + | + | + | + | - | - | - | + | + | + | + | + | + | + | + | + | + | + | + | + | + | + | + | + | + | + | + | + | + | - |
| ABI163 | December 2018 | ICU 5 | Trachea | - | + | + | + | + | - | - | - | + | + | + | + | + | + | + | + | + | + | + | + | + | + | + | + | + | + | + | + | + | - | ND |
| ABI166 | December 2018 | ICU 4 | Trachea | - | + | + | + | + | - | - | - | + | + | + | + | + | + | + | + | + | + | + | + | + | + | + | - | - | - | + | + | + | + | + |
| ABI167 | December 2018 | ICU 1 | Blood | - | + | + | + | + | - | - | - | + | + | + | + | + | + | + | + | + | + | + | + | + | + | + | + | + | + | + | + | + | + | - |
| ABI181 | January 2019 | Ward 15 | Wound | - | + | + | + | + | - | - | - | + | + | + | + | + | + | + | + | + | + | + | + | + | + | + | + | + | + | + | + | + | - | ND |
| ABI184 | January 2019 | Ward 18 | Discharges | - | + | + | + | + | + | + | + | + | + | + | + | + | + | + | + | + | + | + | + | + | + | + | - | - | - | + | + | + | + | + |
| ABI188 | January 2019 | ICU 10 | Discharges | - | + | + | + | - | + | + | + | + | + | + | + | + | + | + | + | + | + | + | + | + | + | + | - | - | - | + | + | + | + | - |
| ABI189 | January 2019 | ICU 4 | Trachea | - | + | + | + | + | - | - | - | + | + | + | + | + | + | + | + | + | + | + | + | + | + | + | - | - | - | + | + | + | + | + |
| ABI192 | January 2019 | ICU 10 | Blood | - | + | + | + | + | + | + | + | + | + | + | + | + | + | + | + | + | + | + | + | + | + | + | + | + | + | + | + | + | + | + |
| ABI193 | January 2019 | ICU 10 | Blood | - | + | + | + | + | - | - | - | + | + | + | + | + | + | + | + | + | + | + | + | + | + | + | + | + | + | + | + | + | + | - |
| ABI195 | June 2019 | ICU 1 | Trachea | - | + | + | + | + | + | + | + | + | + | + | + | + | + | + | + | + | + | + | + | + | + | + | + | + | + | + | + | + | + | - |
| ABI196 | June 2019 | Ward 16 | Sputum | - | + | + | + | - | + | + | + | + | - | - | + | + | + | + | + | + | + | + | + | + | + | + | - | - | - | + | + | + | + | + |
| ABI201 | July 2019 | ICU 2 | Ascites | - | + | + | + | - | + | + | + | + | - | - | + | + | + | + | + | + | + | + | + | + | + | + | - | - | - | + | + | + | + | + |
| ABI203 | July 2019 | Ward 10 | CSF | - | + | + | + | - | + | + | + | + | - | - | + | + | + | + | + | + | + | + | + | + | + | + | - | - | - | + | + | + | + | + |
| ABI205 | July 2019 | ICU 10 | Trachea | - | + | + | + | + | + | + | + | + | + | + | + | + | + | + | + | + | + | + | + | + | + | + | + | + | + | + | + | + | + | + |
| ABI206 | July 2019 | ICU 9 | Trachea | - | + | + | + | - | + | + | + | + | - | - | + | + | + | + | + | + | + | + | + | + | + | + | - | - | - | + | + | + | + | + |
| ABI207 | July 2019 | Ward 21 | Wound | - | + | + | + | - | + | + | + | + | - | - | + | + | + | + | + | + | + | + | + | + | + | + | - | - | - | + | + | + | + | + |
| ABI215 | July 2019 | ICU 10 | Blood | - | + | + | + | - | + | + | + | + | - | - | + | + | + | + | + | + | + | + | + | + | + | + | - | - | - | + | + | + | + | + |
| ABI216 | July 2019 | ICU 8 | Trachea | - | + | + | + | + | + | + | + | + | + | + | + | + | + | + | + | + | + | + | + | + | + | + | + | + | + | + | + | + | - | ND |
| ABI219 | July 2019 | Ward 14 | Cynovial fluid | - | + | + | + | + | - | - | - | + | + | + | + | + | + | + | + | + | + | + | + | + | + | + | + | + | + | + | + | + | + | + |
| ABI220 | July 2019 | Ward 17 | Pericardial effusion | - | + | + | + | + | + | + | + | + | + | + | + | + | + | + | + | + | + | + | + | + | + | + | + | + | + | + | + | + | + | - |
| ABI221 | July 2019 | Ward 2 | Wound | - | + | + | + | + | + | + | + | + | + | + | + | + | + | + | + | + | + | + | + | + | + | + | + | + | + | + | + | + | + | + |
| ABI227 | August 2019 | ICU 2 | Trachea | - | + | + | + | + | + | + | + | + | + | + | + | + | + | + | + | + | + | + | + | + | + | + | + | + | + | + | + | + | - | ND |
| ABI231 | August 2019 | Ward 7 | Wound | - | + | + | + | + | + | + | + | + | + | + | + | + | + | + | + | + | + | + | + | + | + | + | + | + | + | + | + | + | + | + |
| ABI233 | August 2019 | Ward 9 | Blood | - | + | + | + | - | + | + | + | + | - | - | + | + | + | + | + | + | + | + | + | + | + | + | - | - | - | + | + | + | + | + |
| ABI239 | September 2019 | ICU 8 | Trachea | - | + | + | + | + | + | + | + | + | + | + | + | + | + | + | + | + | + | + | + | + | + | + | - | - | - | + | + | + | + | - |
| ABI243 | September 2019 | ICU 10 | Trachea | - | + | + | + | + | + | + | + | + | + | + | + | + | + | + | + | + | + | + | + | + | + | + | + | + | + | + | + | + | + | - |
| ABI248 | October 2019 | ICU 10 | Trachea | - | + | + | + | + | + | + | + | + | + | + | + | + | + | + | + | + | + | + | + | + | + | + | + | + | + | + | + | + | + | + |
| ABI250 | October 2019 | Ward 5 | Sputum | - | + | + | + | + | + | + | + | + | + | + | + | + | + | + | + | + | + | + | + | + | + | + | + | + | + | + | + | + | - | ND |
| ABI251 | October 2019 | Ward 11 | Trachea | - | + | + | + | - | + | + | + | + | - | - | + | + | + | + | + | + | + | + | + | + | + | + | - | - | - | + | + | + | + | + |
| ABI252 | October 2019 | ICU 5 | Trachea | - | + | + | + | + | + | + | + | + | + | + | + | + | + | + | + | + | + | + | + | + | + | + | + | + | + | + | + | + | + | - |
| ABI255 | October 2019 | Ward 12 | Sputum | - | + | + | + | + | + | + | + | + | + | + | + | + | + | + | + | + | + | + | + | + | + | + | + | + | + | + | + | + | + | + |
| ABI256 | November 2019 | Ward 6 | Wound | - | + | + | + | + | + | + | + | + | + | + | + | + | + | + | + | + | + | + | + | + | + | + | + | + | + | + | + | + | + | + |
| ABI257 | November 2019 | Ward 9 | Ascites | - | + | + | + | + | + | + | + | + | + | + | + | + | + | + | + | + | + | + | + | + | + | + | + | + | + | + | + | + | + | + |
| ABI258 | November 2019 | Ward 4 | Discharges | - | + | + | + | + | + | + | + | + | + | + | + | + | + | + | + | + | + | + | + | + | + | + | + | + | + | + | + | + | + | + |
| ABI260 | November 2019 | ICU 9 | Catheter | - | + | + | + | + | + | + | + | + | + | + | + | + | + | + | + | + | + | + | + | + | + | + | + | + | + | + | + | + | - | ND |
| ABI261 | November 2019 | ICU 9 | Discharges | - | + | + | + | + | + | + | + | + | + | + | + | + | + | + | + | + | + | + | + | + | + | + | + | + | + | + | + | + | + | + |
| ABI262 | November 2019 | Ward 19 | Ascites | - | + | + | + | + | + | + | + | + | + | + | + | + | + | + | + | + | + | + | + | + | + | + | + | + | + | + | + | + | + | + |
| ABI263 | November 2019 | ICU 9 | Blood | - | + | + | + | + | + | + | + | + | + | + | + | + | + | + | + | + | + | + | + | + | + | + | - | - | - | + | + | + | + | + |
| ABI265 | November 2019 | Ward 13 | Catheter | - | + | + | + | + | + | + | + | + | + | + | + | + | + | + | + | + | + | + | + | + | + | + | + | + | + | + | + | + | + | - |
| ABI266 | November 2019 | ICU 10 | Trachea | - | + | + | + | + | + | + | + | + | + | + | + | + | + | + | + | + | + | + | + | + | + | + | + | + | + | + | + | + | + | + |
| ABI268 | November 2019 | ICU 9 | Trachea | - | + | + | + | + | + | + | + | + | + | + | + | + | + | + | + | + | + | + | + | + | + | + | + | + | + | + | + | + | - | ND |
| ABI269 | November 2019 | ICU 10 | Blood | - | + | + | + | - | + | + | + | + | + | + | + | + | + | + | + | + | + | + | + | + | + | + | - | - | - | + | + | + | - | ND |
| ABI272 | January 2020 | Ward 8 | Blood | - | + | + | + | - | + | + | + | + | - | - | + | + | + | + | + | + | + | + | + | + | + | + | - | - | - | + | + | + | + | + |
| ABI273 | January 2020 | Ward 9 | Blood | - | + | + | + | + | + | + | + | + | + | + | + | + | + | + | + | + | + | + | + | + | + | + | + | + | + | + | + | + | + | + |
| ABI275 | January 2020 | ICU 10 | Trachea | - | + | + | + | + | - | - | - | + | + | + | + | + | + | + | + | + | + | + | + | + | + | + | + | + | + | + | + | + | - | ND |
| ABI283 | January 2020 | Ward 21 | Wound | - | + | + | + | + | + | + | + | + | + | + | + | + | + | + | + | + | + | + | + | + | + | + | + | + | + | + | + | + | + | + |
| ABI286 | January 2020 | Ward 20 | Discharges | - | + | + | + | + | + | + | + | + | + | + | + | + | + | + | + | + | + | + | + | + | + | + | + | + | + | + | + | + | + | - |

The PCRs in bold are the linkage PCRs that were performed for identification of AbGRI1s in this study.

ND, Not determined.

NR, Not recorded.

**Table S3**. Characteristics of the GC2 isolates containing AbGRI2 and AbGRI3 resistance islands.

| Isolate | Isolation date | Ward | Source | *bla_TEM_* | *aphA1b* | *sul1* | *aadA1* | *aacC1* | *aacC1*-*aadA1* | *intI1* | IS*26*-*aphA1* | *aphA1*-IS*26* | AB57_1175 - t*npR_1_* | *bla_TEM-_ tnpA_1000_* | *tnpR_5393_c- aphA1* | *aphA1- sul1* | tnpA_21_-AB57_1209 | TE32_13140-*tnpR1* | *aphA1b*-ABA1_01228 | ***tnpR_5393_c-* ABA1_01228** | AbGRI2 | *armA* | *aacA4* | *atr* | *Δatr-repAciN* | *aphA1b-Δasr* | *intI1-aphA1b* | *intI1-Δasr* | *atrΔ-asrΔ* | *armA-asrΔ* | *Δatr-I*SAba24 | AbGRI3 |
| --- | --- | --- | --- | --- | --- | --- | --- | --- | --- | --- | --- | --- | --- | --- | --- | --- | --- | --- | --- | --- | --- | --- | --- | --- | --- | --- | --- | --- | --- | --- | --- | --- |
| ABI001 | November 2011 | ICU 10 | Blood | - | - | - | - | - | ND | ND | - | - | - | - | - | - | - | ND | ND | ND | No AbGRI2 | + | - | - | + | ND | ND | ND | ND | + | ND | AbGRI3-4 |
| ABI002 | November 2011 | ICU 10 | Blood | - | - | - | - | - | ND | ND | - | - | - | - | - | - | - | ND | ND | ND | No AbGRI2 | - | - | + | ND | ND | ND | ND | ND | ND | ND | No AbGRI3 |
| ABI004 | November 2011 | Ward 21 | Catheter | + | + | - | - | - | ND | ND | + | + | - | + | + | - | - | + | + | ND | AbGRI2-12a | + | - | - | + | ND | ND | ND | ND | + | ND | AbGRI3-4 |
| ABI005 | November 2011 | Ward 13 | Ascites | + | + | - | - | - | ND | ND | + | + | - | + | + | - | - | + | + | ND | AbGRI2-12b | + | - | - | + | ND | ND | ND | ND | + | ND | AbGRI3-4 |
| ABI007 | November 2011 | ICU 10 | Blood | + | + | - | - | - | ND | ND | + | + | - | + | + | - | - | + | + | ND | AbGRI2-12b | - | - | + | ND | ND | ND | ND | ND | ND | ND | No AbGRI3 |
| ABI014 | December 2011 | NR | Trachea | + | + | - | - | - | ND | ND | + | + | - | + | + | - | - | + | + | ND | AbGRI2-12b | + | - | - | + | ND | ND | ND | ND | + | ND | AbGRI3-4 |
| ABI020 | December 2011 | ICU 10 | Trachea | - | - | - | - | - | ND | ND | - | - | - | - | - | - | - | ND | ND | ND | No AbGRI2 | + | - | - | + | ND | ND | ND | ND | + | ND | AbGRI3-4 |
| ABI024 | January 2012 | Ward 5 | Trachea | - | - | - | - | - | ND | ND | - | - | - | - | - | - | - | ND | ND | ND | No AbGRI2 | - | - | + | ND | ND | ND | ND | ND | ND | ND | No AbGRI3 |
| ABI026 | January 2012 | Ward 5 | NR | + | + | - | - | - | ND | ND | + | + | - | + | + | - | - | + | + | ND | AbGRI2-12b | + | - | - | + | ND | ND | ND | ND | + | ND | AbGRI3-4 |
| ABI028 | April 2012 | NR | Trachea | + | + | - | - | - | ND | ND | + | + | - | + | + | - | - | + | + | ND | AbGRI2-12b | + | - | - | + | ND | ND | ND | ND | + | ND | AbGRI3-4 |
| ABI034 | January 2012 | ICU 10 | Wound | - | - | - | - | - | ND | ND | - | - | - | - | - | - | - | ND | ND | ND | No AbGRI2 | + | - | - | + | ND | ND | ND | ND | + | ND | AbGRI3-4 |
| ABI045 | May 2012 | NR | Wound | + | + | + | + | + | + | + | + | + | + | + | + | + | + | ND | ND | ND | AbGRI2-1 | - | - | + | ND | ND | ND | ND | ND | ND | ND | No AbGRI3 |
| ABI046 | May 2012 | NR | Wound | + | + | - | - | - | ND | ND | + | + | - | + | + | - | - | + | + | ND | AbGRI2-12b | + | - | - | + | ND | ND | ND | ND | + | ND | AbGRI3-4 |
| ABI052 | June 2012 | NR | NR | - | - | - | - | - | ND | ND | - | - | - | - | - | - | - | ND | ND | ND | No AbGRI2 | - | - | + | ND | ND | ND | ND | ND | ND | ND | No AbGRI3 |
| ABI076 | September 2012 | NR | NR | + | + | - | - | - | ND | ND | + | + | - | + | + | - | - | + | + | ND | AbGRI2-12b | + | - | - | + | ND | ND | ND | ND | + | ND | AbGRI3-4 |
| ABI143 | December 2018 | ICU 3 | Trachea | + | + | - | - | - | ND | ND | + | + | - | + | + | - | - | + | + | ND | AbGRI2-12b | + | - | - | + | ND | ND | ND | ND | + | ND | AbGRI3-4 |
| ABI151 | January 2019 | ICU 1 | BAL | + | - | - | - | - | ND | ND | - | - | - | + | - | - | - | + | - | + | AbGRI2_ABI257_ | + | - | - | + | ND | ND | ND | ND | + | ND | AbGRI3-4 |
| ABI154 | December 2018 | ICU 2 | Discharges | - | - | - | - | - | ND | ND | - | - | - | - | - | - | - | ND | ND | ND | No AbGRI2 | - | - | + | ND | ND | ND | ND | ND | ND | ND | No AbGRI3 |
| ABI157 | December 2018 | ICU 10 | Trachea | - | - | - | - | - | ND | ND | - | - | - | - | - | - | - | ND | ND | ND | No AbGRI2 | - | - | + | ND | ND | ND | ND | ND | ND | ND | No AbGRI3 |
| ABI159 | December 2018 | Ward 5 | CSF | - | - | - | - | - | ND | ND | - | - | - | - | - | - | - | ND | ND | ND | No AbGRI2 | + | - | - | + | ND | ND | ND | ND | + | ND | AbGRI3-4 |
| ABI163 | December 2018 | ICU 5 | Trachea | - | - | - | - | - | ND | ND | - | - | - | - | - | - | - | ND | ND | ND | No AbGRI2 | - | - | + | ND | ND | ND | ND | ND | ND | ND | No AbGRI3 |
| ABI166 | December 2018 | ICU 4 | Trachea | + | + | - | - | - | ND | ND | + | + | - | + | + | - | - | + | + | ND | AbGRI2-12b | + | - | - | + | ND | ND | ND | ND | + | ND | AbGRI3-4 |
| ABI167 | December 2018 | ICU 1 | Blood | - | - | - | - | - | ND | ND | - | - | - | - | - | - | - | ND | ND | ND | No AbGRI2 | - | - | + | ND | ND | ND | ND | ND | ND | ND | No AbGRI3 |
| ABI169 | December 2018 | ICU 5 | Blood | + | + | - | - | - | ND | ND | + | + | - | + | + | - | - | + | + | ND | AbGRI2-12b | + | - | - | + | ND | ND | ND | ND | + | ND | AbGRI3-4 |
| ABI171 | December 2018 | ICU 7 | Blood | - | - | - | - | - | ND | ND | - | - | - | - | - | - | - | ND | ND | ND | No AbGRI2 | + | - | - | + | ND | ND | ND | ND | + | ND | AbGRI3-4 |
| ABI179 | January 2019 | Ward 1 | Wound | + | - | - | - | - | ND | ND | - | - | - | + | - | - | - | + | - | + | AbGRI2_ABI257_ | + | - | - | + | ND | ND | ND | ND | + | ND | AbGRI3-4 |
| ABI181 | January 2019 | Ward 15 | Wound | - | - | - | - | - | ND | ND | - | - | - | - | - | - | - | ND | ND | ND | No AbGRI2 | - | - | + | ND | ND | ND | ND | ND | ND | ND | No AbGRI3 |
| ABI184 | January 2019 | Ward 18 | Discharges | + | + | + | + | + | + | + | + | + | + | + | + | + | + | ND | ND | ND | AbGRI2-1 | - | - | + | ND | ND | ND | ND | ND | ND | ND | No AbGRI3 |
| ABI185 | January 2019 | ICU 9 | Trachea | - | - | - | - | - | ND | ND | - | - | - | - | - | - | - | ND | ND | ND | No AbGRI2 | + | - | - | + | ND | ND | ND | ND | + | ND | AbGRI3-4 |
| ABI188 | January 2019 | ICU 10 | Discharges | + | + | + | + | + | + | + | + | + | + | + | + | + | + | ND | ND | ND | AbGRI2-1 | - | - | + | ND | ND | ND | ND | ND | ND | ND | No AbGRI3 |
| ABI189 | January 2019 | ICU 4 | Trachea | + | + | - | - | - | ND | ND | + | + | - | + | + | - | - | + | + | ND | AbGRI2-12b | + | - | - | + | ND | ND | ND | ND | + | ND | AbGRI3-4 |
| ABI190 | January 2019 | ICU 1 | Trachea | - | - | - | - | - | ND | ND | - | - | - | - | - | - | - | ND | ND | ND | No AbGRI2 | + | - | - | + | ND | ND | ND | ND | + | ND | AbGRI3-4 |
| ABI191 | February 2019 | Ward 2 | Blood | + | + | - | - | - | ND | ND | + | + | - | + | + | - | - | + | + | ND | AbGRI2-12b | + | - | - | + | ND | ND | ND | ND | + | ND | AbGRI3-4 |
| ABI192 | January 2019 | ICU 10 | Blood | + | + | - | - | - | ND | ND | + | + | - | + | + | - | - | + | + | ND | AbGRI2-12b | + | - | - | + | ND | ND | ND | ND | + | ND | AbGRI3-4 |
| ABI193 | January 2019 | ICU 10 | Blood | + | + | - | - | - | ND | ND | + | + | - | + | + | - | - | + | + | ND | AbGRI2-12b | + | - | - | + | ND | ND | ND | ND | + | ND | AbGRI3-4 |
| ABI194 | June 2019 | Ward 3 | Ascites | + | + | - | - | - | ND | ND | + | + | - | + | + | - | - | + | + | ND | AbGRI2-12a | + | - | - | + | ND | ND | ND | ND | + | ND | AbGRI3-4 |
| ABI195 | June 2019 | ICU 1 | Trachea | + | + | + | + | + | + | + | + | + | + | + | + | + | + | ND | ND | ND | AbGRI2-1 | - | - | + | ND | ND | ND | ND | ND | ND | ND | No AbGRI3 |
| ABI196 | June 2019 | Ward 16 | Sputum | - | - | - | - | - | ND | ND | - | - | - | - | - | - | - | ND | ND | ND | No AbGRI2 | - | - | + | ND | ND | ND | ND | ND | ND | ND | No AbGRI3 |
| ABI198 | June 2019 | ICU 6 | Trachea | + | + | - | - | - | ND | ND | + | + | - | + | + | - | - | + | + | ND | AbGRI2-12b | + | - | - | + | ND | ND | ND | ND | + | ND | AbGRI3-4 |
| ABI199 | June 2019 | Ward 4 | Pleural effusion | + | + | - | - | - | ND | ND | + | + | - | + | + | - | - | + | + | ND | AbGRI2-12a | + | - | - | + | ND | ND | ND | ND | + | ND | AbGRI3-4 |
| ABI201 | July 2019 | ICU 2 | Ascites | - | - | - | - | - | ND | ND | - | - | - | - | - | - | - | ND | ND | ND | No AbGRI2 | + | - | - | + | ND | ND | ND | ND | + | ND | AbGRI3-4 |
| ABI203 | July 2019 | Ward 10 | CSF | - | - | - | - | - | ND | ND | - | - | - | - | - | - | - | ND | ND | ND | No AbGRI2 | + | - | - | - | ND | ND | ND | ND | + | + | AbGRI3_ABI221_ |
| ABI204 | July 2019 | ICU 10 | Trachea | - | - | - | - | - | ND | ND | - | - | - | - | - | - | - | ND | ND | ND | No AbGRI2 | + | - | - | + | ND | ND | ND | ND | + | ND | AbGRI3-4 |
| ABI205 | July 2019 | ICU 10 | Trachea | - | - | - | - | - | ND | ND | - | - | - | - | - | - | - | ND | ND | ND | No AbGRI2 | + | - | - |  | ND | ND | ND | ND |  |  | AbGRI3_ABI221_ |
| ABI206 | July 2019 | ICU 9 | Trachea | - | - | - | - | - | ND | ND | - | - | - | - | - | - | - | ND | ND | ND | No AbGRI2 | + | - | - | + | ND | ND | ND | ND | + | ND | AbGRI3-4 |
| ABI207 | July 2019 | Ward 21 | Wound | + | + | - | - | - | ND | ND | + | + | - | + | + | - | - | + | + | ND | AbGRI2-12b | + | - | - | + | ND | ND | ND | ND | + | ND | AbGRI3-4 |
| ABI215 | July 2019 | ICU 10 | Blood | - | - | - | - | - | ND | ND | - | - | - | - | - | - | - | ND | ND | ND | No AbGRI2 | + | - | - | + | ND | ND | ND | ND | + | ND | AbGRI3-4 |
| ABI216 | July 2019 | ICU 8 | Trachea | - | - | - | - | - | ND | ND | - | - | - | - | - | - | - | ND | ND | ND | No AbGRI2 | + | - | - | - | ND | ND | ND | ND | + | + | AbGRI3_ABI221_ |
| ABI219 | July 2019 | Ward 14 | Cynovial fluid | - | - | - | - | - | ND | ND | - | - | - | - | - | - | - | ND | ND | ND | No AbGRI2 | + | - | - | - | ND | ND | ND | ND | + | + | AbGRI3_ABI221_ |
| ABI220 | July 2019 | Ward 17 | Pericardial effusion | - | - | - | - | - | ND | ND | - | - | - | - | - | - | - | ND | ND | ND | No AbGRI2 | - | - | + | ND | ND | ND | ND | ND | ND | ND | No AbGRI3 |
| ABI221 | July 2019 | Ward 2 | Wound | + | - | - | - | - | ND | ND | - | - | - | + | - | - | - | + | - | + | AbGRI2_ABI257_ | + | - | - | - | ND | ND | ND | ND | + | + | AbGRI3_ABI221_ |
| ABI222 | July 2019 | ICU 7 | Discharges | + | + | - | - | - | ND | ND | + | + |  | + | + |  |  | + | + | ND | AbGRI2-12b | + | - | - | + | ND | ND | ND | ND | + | ND | AbGRI3-4 |
| ABI223 | July 2019 | ICU 3 | Trachea | + | - | - | - | - | ND | ND | - | - | - | + | - | - | - | + | - | + | AbGRI2_ABI257_ | + | - | - | + | ND | ND | ND | ND | + | ND | AbGRI3-4 |
| ABI227 | August 2019 | ICU 2 | Trachea | - | - | - | - | - | ND | ND | - | - | - | - | - | - | - | ND | ND | ND | No AbGRI2 | + | - | - | + | ND | ND | ND | ND | + | ND | AbGRI3-4 |
| ABI229 | August 2019 | Ward 7 | Wound | + | + | - | - | - | ND | ND | + | + | - | - | + | - | - | + | + | ND | AbGRI2-12b | - | - | + | ND | ND | ND | ND | ND | ND | ND | No AbGRI3 |
| ABI230 | August 2019 | Ward 3 | Trachea | - | - | - | - | - | ND | ND | - | - | - | - | - | - | - | ND | ND | ND | No AbGRI2 | + | - | - | + | ND | ND | ND | ND | + | ND | AbGRI3-4 |
| ABI231 | August 2019 | Ward 7 | Wound | - | - | - | - | - | ND | ND | - | - | - | - | - | - | - | ND | ND | ND | No AbGRI2 | + | - | - | + | ND | ND | ND | ND | + | ND | AbGRI3-4 |
| ABI233 | August 2019 | Ward 9 | Blood | - | - | - | - | - | ND | ND | - | - | - | - | - | - | - | ND | ND | ND | No AbGRI2 | + | - | - | + | ND | ND | ND | ND | + | ND | AbGRI3-4 |
| ABI239 | September 2019 | ICU 8 | Trachea | - | - | - | - | - | ND | ND | - | - | - | - | - | - | - | ND | ND | ND | No AbGRI2 | + | - | - | - | ND | ND | ND | ND | + | + | AbGRI3_ABI221_ |
| ABI242 | September 2019 | ICU 10 | Trachea | - | - | - | - | - | ND | ND | - | - | - | - | - | - | - | ND | ND | ND | No AbGRI2 | + | - | - | + | ND | ND | ND | ND | + | ND | AbGRI3-4 |
| ABI243 | September 2019 | ICU 10 | Trachea | + | - | - | - | - | ND | ND | - | - | - | + | - | - | - | + | - | + | AbGRI2_ABI257_ | + | - | - | - | ND | ND | ND | ND | + | + | AbGRI3_ABI221_ |
| ABI248 | October 2019 | ICU 10 | Trachea | + | + | - | - | - | ND | ND | + | + | - | + | + | - | - | + | + | ND | AbGRI2-12b | + | - | - | + | ND | ND | ND | ND | + | ND | AbGRI3-4 |
| ABI249 | October 2019 | ICU 1 | Discharges | + | + | - | - | - | ND | ND | + | + | - | + | + | - | - | + | + | ND | AbGRI2-12b | + | - | - | + | ND | ND | ND | ND | + | ND | AbGRI3-4 |
| ABI250 | October 2019 | Ward 5 | Sputum | - | - | - | - | - | ND | ND | - | - | - | - | - | - | - | ND | ND | ND | No AbGRI2 | + | - | - | + | ND | ND | ND | ND | + | ND | AbGRI3-4 |
| ABI251 | October 2019 | Ward 11 | Trachea | - | - | - | - | - | ND | ND | - | - | - | - | - | - | - | ND | ND | ND | No AbGRI2 | + | - | - | - | ND | ND | ND | ND | + | + | AbGRI3_ABI221_ |
| ABI252 | October 2019 | ICU 5 | Trachea | + | + | - | - | - | ND | ND | + | + | - | + | + | - | - | + | + | ND | AbGRI2-12b | + | - | - | + | ND | ND | ND | ND | + | ND | AbGRI3-4 |
| ABI254 | October 2019 | ICU 8 | Trachea | + | + | - | - | - | ND | ND | + | + | - | + | + | - | - | + | + | ND | AbGRI2-12a | + | - | - | + | ND | ND | ND | ND | + | ND | AbGRI3-4 |
| ABI255 | October 2019 | Ward 12 | Sputum | - | - | - | - | - | ND | ND | - | - | - | - | - | - | - | ND | ND | ND | No AbGRI2 | + | - | - | - | ND | ND | ND | ND | + | + | AbGRI3_ABI221_ |
| ABI256 | November 2019 | Ward 6 | Wound | + | + | - | - | - | ND | ND | + | + | - | + | + | - | - | + | + | ND | AbGRI2-12a | + | - | - | + | ND | ND | ND | ND | + | ND | AbGRI3-4 |
| ABI257 | November 2019 | Ward 9 | Ascites | + | - | - | - | - | ND | ND | - | - | - | + | - | - | - | + | - | + | AbGRI2_ABI257_ | + | - | - | - | ND | ND | ND | ND | + | + | AbGRI3_ABI221_ |
| ABI258 | November 2019 | Ward 4 | Discharges | + | - | - | - | - | ND | ND | - | - | - | + | - | - | - | + | - | + | AbGRI2_ABI257_ | + | - | - | - | ND | ND | ND | ND | + | + | AbGRI3_ABI221_ |
| ABI259 | November 2019 | ICU 5 | Sputum | + | + | - | - | - | ND | ND | + | + | - | - | + | - | - | + | + | ND | AbGRI2-12a | + | - | - | + | ND | ND | ND | ND | + | ND | AbGRI3-4 |
| ABI260 | November 2019 | ICU 9 | Catheter | - | - | - | - | - | ND | ND | - | - | - | - | - | - | - | ND | ND | ND | No AbGRI2 | + | - | - | - | ND | ND | ND | ND | + | + | AbGRI3_ABI221_ |
| ABI261 | November 2019 | ICU 9 | Discharges | - | - | - | - | - | ND | ND | - | - | - | - | - | - | - | ND | ND | ND | No AbGRI2 | + | - | - | + | ND | ND | ND | ND | + | ND | AbGRI3-4 |
| ABI262 | November 2019 | Ward 19 | Ascites | + | - | - | - | - | ND | ND | - | - | - | + | - | - | - | + | - | + | AbGRI2_ABI257_ | + | - | - | + | ND | ND | ND | ND | + | ND | AbGRI3-4 |
| ABI263 | November 2019 | ICU 9 | Blood | + | - | - | - | - | ND | ND | - | - | - | + | - | - | - | + | - | + | AbGRI2_ABI257_ | + | - | - | - | ND | ND | ND | ND | + | + | AbGRI3_ABI221_ |
| ABI265 | November 2019 | Ward 13 | Catheter | - | - | - | - | - | ND | ND | - | - | - | - | - | - | - | ND | ND | ND | No AbGRI2 | + | - | - | - | ND | ND | ND | ND | + | + | AbGRI3_ABI221_ |
| ABI266 | November 2019 | ICU 10 | Trachea | + | - | - | - | - | ND | ND | - | - | - | + | - | - | - | + | - | + | AbGRI2_ABI257_ | + | - | - | + | ND | ND | ND | ND | + | ND | AbGRI3-4 |
| ABI268 | November 2019 | ICU 9 | Trachea | + | - | - | - | - | ND | ND | - | - | - | + | - | - | - | + | - | + | AbGRI2_ABI257_ | + | - | - | + | ND | ND | ND | ND | + | ND | AbGRI3-4 |
| ABI269 | November 2019 | ICU 10 | Blood | + | + | + | + | + | + | + | + | + | + | + | + | + | + | ND | ND | ND | AbGRI2-1 | - | - | + | ND | ND | ND | ND | ND | ND | ND | No AbGRI3 |
| ABI270 | December 2019 | ICU 10 | Blood | + | - | - | - | - | ND | ND | - | - | - | + | - | - | - | + | - | + | AbGRI2_ABI257_ | + | - | - | + | ND | ND | ND | ND | + | ND | AbGRI3-4 |
| ABI271 | December 2019 | ICU 4 | Blood | + | - | - | - | - | ND | ND | - | - | - | + | - | - | - | + | - | + | AbGRI2_ABI257_ | + | - | - | + | ND | ND | ND | ND | + | ND | AbGRI3-4 |
| ABI272 | January 2020 | Ward 8 | Blood | - | - | - | - | - | ND | ND | - | - | - | - | - | - | - | ND | ND | ND | No AbGRI2 | + | - | - | + | ND | ND | ND | ND | + | ND | AbGRI3-4 |
| ABI273 | January 2020 | Ward 9 | Blood | + | + | - | - | - | ND | ND | + | + | - | + | + | - | - | + | + | ND | AbGRI2-12a | + | - | - | + | ND | ND | ND | ND | + | ND | AbGRI3-4 |
| ABI275 | January 2020 | ICU 10 | Trachea | - | - | - | - | - | ND | ND | - | - | - | - | - | - | - | ND | ND | ND | No AbGRI2 | - | - | + | ND | ND | ND | ND | ND | ND | ND | No AbGRI3 |
| ABI277 | January 2020 | ICU 10 | Trachea | + | - | - | - | - | ND | ND | - | - | - | + | - | - | - | + | - | + | AbGRI2_ABI257_ | + | - | - | - | ND | ND | ND | ND | + | + | AbGRI3_ABI221_ |
| ABI281 | January 2020 | ICU 10 | Sputum | + | - | - | - | - | ND | ND | - | - | - | + | - | - | - | + | - | + | AbGRI2_ABI257_ | + | - | - | + | ND | ND | ND | ND | + | ND | AbGRI3-4 |
| ABI283 | January 2020 | Ward 21 | Wound | + | - | - | - | - | ND | ND | - | - | - | + | - | - | - | + | - | + | AbGRI2_ABI257_ | + | - | - | - | ND | ND | ND | ND | + | + | AbGRI3_ABI221_ |
| ABI284 | January 2020 | ICU 10 | Trachea | - | - | - | - | - | ND | ND | - | - | - | - | - | - | - | ND | ND | ND | No AbGRI2 | + | - | - | + | ND | ND | ND | ND | + | ND | AbGRI3-4 |
| ABI286 | January 2020 | Ward 20 | Discharges | + | - | - | - | - | ND | ND | - | - | - | + | - | - | - | + | - | + | AbGRI2_ABI257_ | + | - | - | + | ND | ND | ND | ND | + | ND | AbGRI3-4 |

The PCR in bold is a new linkage PCR that was performed for identification of AbGRI2 in this study.

CSF, Cerebrospinal fluid; BAL, Bronchoalveolar lavage.

ND, Not determined.

NR, Not recorded.

**Table S4**. The backbone transposon and *sul2* gene profiles in GC2 *A. baumannii* isolates containing AbGRI1 resistance island

| Group | *tniB∆-tniE∆* | *tniB-tniE* | *sul2* | Isolates |
| --- | --- | --- | --- | --- |
| 1 | + | + | + | ABI001, ABI004, ABI005, ABI014, ABI020, ABI026, ABI028, ABI034, ABI046, ABI076, ABI143, ABI184, ABI192, ABI195, ABI205, ABI216, ABI220, ABI221, ABI227, ABI231, ABI239, ABI243, ABI248, ABI250, ABI252, ABI255, ABI256, ABI257, ABI258, ABI260, ABI261, ABI262, ABI263, ABI265, ABI266, ABI268, ABI273, ABI283, ABI286. |
| 2 | + | - | + | ABI002, ABI007, ABI024, ABI052, ABI154, ABI157, ABI163, ABI166, ABI167, ABI181, ABI189, ABI193, ABI219, ABI275. |
| 3 | - | + | + | ABI188, ABI269, ABI045. |
| 4 | - | + | - | ABI196, ABI201, ABI203, ABI206, ABI207, ABI215, ABI233, ABI251, ABI272. |

**Table S5**. Primer pairs used for mapping of AbGRI resistance islands.

| **PCR** | **Primer** | **Sequence (5'-3')** | **Annealing**  **temperature**  **(° C)** | **Amplicon length**  **(bp)** | **Reference** |
| --- | --- | --- | --- | --- | --- |
| *comM* | RH927  RH928 | CAACCCTGTCTTTGCATTTG  GCCAGCAAGCTCAGCATAA | 59 | 880 | (21) |
| *comM*–AbGRI1  (J1) | RH927  RH792 | CAACCCTGTCTTTGCATTTG  TTCGAGCTTGAAAACTGCAC | 60 | 846 | (31) |
| AbGRI1–*comM*  (J2) | RH928  RH916 | GCCAGCAAGCTCAGCATAA  CCCAAATACTGCCATGTTGA | 60 | 796 | (31) |
| orf4b-*comM* | RH594  RH928 | GGCGGATTATCAGTTGTTTCA  GCCAGCAAGCTCAGCATAA | 60 | 1844 | (21) |
| *tniBΔ*-*tniEΔ* | RH910  RH587 | GCGATAGTGAACGGATTGAGA  TTGCCCATTAAGCACAACAG | 60 | 560 | (32) |
| *tniE*-*tniB* | RH910  RH587 | GCGATAGTGAACGGATTGAGA  TTGCCCATTAAGCACAACAG | 60 | 3410 | (32) |
| *tniD*-*tniB* | RH910  RH584 | GCGATAGTGAACGGATTGAGA  TCAATATGCCTCGCTCCACT | 60 | 2010 | (21) |
| *uspA*- *tniD* | RH583  RH919 | TCCTGTCTCTCGTGTAGCAAT  TGTCAAAAATTATTGCATGT | 60 | 3577 | (21) |
| Tn-*comM* | RH791  RH909 | TGCTGCAATGAGCTGAAAGT  GCGATTCAAAATATCGGTCAA | 60 | 3119 | (31) |
| *uspA* | RH919  RH793 | TGTCAAAAATTATTGCATGT  CCCAAGAGAGCTGATTTTGC | 58 | 632 | (31) |
| *sup* | RH2523  RH2509 | CCCACTTTAGGATCAACGCC  GTGGTGTAGTCGCTTGTGTG | 60 | 209 | (8) |
| *uspA-sup* | RH793  RH771 | CCCAAGAGAGCTGATTTTGC  TGTAAAATCTGGTGGTCGTAC | 60 | 3267 | (21) |
| *sul2* | sul2-F  sul2-R | GGCAGATGTGATCGACCTCG  ATGCCGGGATCAAGGACAAG | 60 | 407 | (33) |
| ISAba1*-sul2* | ISAba1B  sul2-R | CATGTAAACCAATGCTCACC  ATGCCGGGATCAAGGACAAG | 60 | 1125 | (21) |
| *strA* | strA-F strA-R | CTTGGTGATAACGGCAATTC  CCAATCGCAGATAGAAGGC | 58 | 548 | (34) |
| *strB* | strB-F  strB-R | ATCGTCAAGGGATTGAAACC  GGATCGTAGAACATATTGGC | 58 | 509 | (34) |
| *strA-strB* | strA-F  strB-R | CTTGGTGATAACGGCAATTC  GGATCGTAGAACATATTGGC | 58 | 1190 | (34) |
| *strA-comM* | strA-R  RH928 | CCAATCGCAGATAGAAGGC  GCCAGCAAGCTCAGCATAA | 60 | 3509 | (21) |
| *strB-*orf4b | strB-R  RH599 | GGATCGTAGAACATATTGGC  ATACTGTTTCAAAAACTGATGAA | 60 | 2620 | (21) |
| CR2 | LECR2  RECR2 | CACTGGCTGGCAATGTCTAG  CTTTGGACCGCAGTTGACTC | 60 | 1793 | (21) |
| **CR2*-* *strB*** | strB-F  RECR2 | ATCGTCAAGGGATTGAAACC  CTTTGGACCGCAGTTGACTC | 60 | 2962 | (21) |
| *tetA(B)* | tetB-F  tetB-R | TTGGTTAGGGGCAAGTTTTG  GTAATGGGCCAATAACACCG | 60 | 658 | (35) |
| *tetR(B)* | RH892  RH893 | ACAGCGCATTAGAGCTGCTT  AGAAGGCTGGCTCTGCACCT | 60 | 528 | (21) |
| ***tetA(B)- tetR(B)*** | tetB-R  RH893 | GTAATGGGCCAATAACACCG  AGAAGGCTGGCTCTGCACCT | 60 | 1693 | (21) |
| *tetA(B)-strB* | tetB-R  strBout | GTAATGGGCCAATAACACCG  AGAGGAGCAACGCGATCTAGC | 60 | 4616 | (21) |
| ***tetR(B)-*CR2** | RH892  LECR2 | ACAGCGCATTAGAGCTGCTT  CACTGGCTGGCAATGTCTAG | 60 | 2812 | (21) |
| orf6-orf7 | RH1302  RH1303 | CAAATCGGGAAGGTTCAAAA  CGGGAAAATTACTGCGATTG | 60 | 1573 | (21) |
| *int*-orf11 | RH1306  RH1307 | GCATACTCATGTGGTTTAAGACTTG  TTAATTGCTTCATCATTTGAGC | 60 | 1638 | (21) |
| orf9-*tniCb* | RH597  RH792 | TTTGAAGAAATTGAGCATGAGG  TTCGAGCTTGAAAACTGCAC | 60 | 1566 | (21) |
| *oxa23* | oxa23F  oxa23R | GATCGGATTGGAGAACCAGA  ATTTCTGACCGCATTTCCAT | 52 | 501 | (36) |
| ISAba1-*oxa23* | ISAba1B  oxa23R | CATGTAAACCAATGCTCACC  ATTTCTGACCGCATTTCCAT | 60 | 1369 | (36, 37) |
| *oxa23*-ISAba1 | oxa23F  ISAba1B | GATCGGATTGGAGAACCAGA  CATGTAAACCAATGCTCACC | 60 | 2725 | (36, 37) |
| *oxa23*-*tetA(B)* | oxa23R  tetBF | ATTTCTGACCGCATTTCCAT  TTGGTTAGGGGCAAGTTTTG | 60 | 5205 | (35, 36) |
| *tniB*-Tn*2006* | RH910  RH743 | GCGATAGTGAACGGATTGAGA  GGCTTCTTGTGGATGCAACT | 60 | 9363 ^a^ | (8) |
| AB57_1175 - t*npR_1_* | RH1315  RH539 | AGGAGATCTTCTTGGCAGTCA  CCAGCCCTTCCCGATCTGTTG | 60 | 1051 | (6) |
| *bla_TEM-_ tnpA_1000_* | RH605  RH759 | TTTCGTGTCGCCCTTATTCC  GCCAGCTCATTTACCTTGCCGA | 60 | 2650 | (6) |
| *tnpR_5393_c-aphA1* | RH520  RH880 | CATGGCCCAGCGCGATACTTCAG  CAACGGGAAACGTCTTGCTC | 60 | 2297 | (6) |
| *aphA1- sul1* | RH881  RH751 | ATTCGTGATTGCGCCTGAG  GCGGAACTTCACGCGATC | 60 | 2712 | (6) |
| *tnpA_21_-*AB57_1209 | RH668  RH1316 | CACCAGAACCGCCTGCTCAA  CATCTGCCATCCAGTTTGTG | 60 | 1219 | (6) |
| TE32_13140-*tnpR1* | RH1563  RH539 | ATAGATCGGCTTCGGACTCA  CCAGCCCTTCCCGATCTGTTG | 60 | 1046 | (8) |
| *aphA1b*-ABA1_01228 | RH881  RH2008 | ATTCGTGATTGCGCCTGAG  TGATGACTTCCATTAAAGCCTGT | 60 | 1581^b^ | (8) |
| ***tnpR_5393_c-* ABA1_01228** | RH520  RH2008 | CATGGCCCAGCGCGATACTTCAG  TGATGACTTCCATTAAAGCCTGT | 60 | 1700 | (8) |
| *aacC1* | RH935  RH936 | GCAGTCGCCCTAAAACAAAG  CCCGTATGCCCAACTTTGTA | 60 | 457 | (21) |
| *aadA1* | RH522  RH531 | GTGGATGGCGGCCTGAAGCCA  GGCAGCGACATCCTTCGGCGC | 60 | 516 | (21) |
| *aacC1-aadA1* | RH935  RH531 | GCAGTCGCCCTAAAACAAAG  GGCAGCGACATCCTTCGGCGC | 60 | 2170^c^ | (21) |
| *bla_TEM_* | RH605  RH606 | TTTCGTGTCGCCCTTATTCC  CCGGCTCCAGATTTATCAGC | 60 | 690 | (21) |
| *sul1* | HS549  HS550 | ACTAAGCTTGCCCCTTCCGC  CTAGGCATGATCTAACCCTCG | 60 | 1100 | (38) |
| *aphA1b* | RH880  RH881 | CAACGGGAAACGTCTTGCTC  ATTCGTGATTGCGCCTGAG | 60 | 454 | (39) |
| IS*26-aphA1b* | RH601  RH880 | GATGGAGCTGCACATGAACC  CAACGGGAAACGTCTTGCTC | 60 | 2121 | (21) |
| *aphA1b-*IS*26* | RH881  IS26F | ATTCGTGATTGCGCCTGAG  ACCTTTGATGGTGGCGTAAG | 60 | 1199 | (21) |
| *armA* | RH2012  RH2013 | TCCATTCCCTTCTCCTTTCC  GGGGGTCTTACTATTCTGCCTA | 60 | 508 | (8) |
| *atr* | RH2001  RH2004 | GGAGTTGGTTTTGGTACAGCA  AATGTGGTTGGCGGTTTTTA | 60 | 400 | (8) |
| *Δatr-repAciN* | RH2001  RH2002 | GGAGTTGGTTTTGGTACAGCA  TATAAGCCACCTCGCTCACC | 60 | 1323 | (7) |
| *aphA1b-Δasr* | RH831  RH2005 | TATACCCATATAAATCAGCATCC  CACTGATCTGCTGGCTTTCA | 60 | 1203 | (7) |
| *armA-asrΔ* | RH2012  RH2014 | TCCATTCCCTTCTCCTTTCC  CCAAATACCGCCCACTCAAC | 60 | 1934 | (7) |
| *Δatr-*ISAba24 | RH2001  RH2010 | GGAGTTGGTTTTGGTACAGCA  TTTCGTGACACTCTCGCTTG | 60 | 1605^d^ | (7) |
| *intI1-aphA1b* | RH2003  RH880 | GCCTTGATGTTACCCGAGAG  CAACGGGAAACGTCTTGCTC | 60 | 1943 | (7) |
| *intI1-Δasr* | RH2003  RH2005 | GCCTTGATGTTACCCGAGAG  CACTGATCTGCTGGCTTTCA | 60 | 1193 | (7) |
| *atrΔ-asrΔ* | RH2015  RH2006 | CCCAGCAATCCATTCGTAGT  TGACGAGCTTTGTTTAGGTGTG | 60 | 1524 | (7) |
| *aacA4* | RH532  RH533 | GTTAGGCATCACAAAGTACAGC  CATCTGGGGTGGTTACGGTACC | 60 | 518 | (39) |

PCRs in bold are the linkage PCRs that were performed for the identification of AbGRI resistance islands in this study.

a. Predicted amplicon size based on AbaR4. For AbaR4∆1 it is 6,513 bp.

b. Predicted size based on AbGRI2-12a. For AbGRI2-12b it is 1,772.

c. Predicted size based on *aacC1*-orfP-orfQ-*aadA1* cassette array. For *aacC1*-orfP-orfP-orfQ-*aadA1* cassette array it is 1,772.

d The size of this segment in the isolates containing AbGRI3_ABI221_ is 3800 bp.

REFERENCES

31. Hamidian M, Hall RM. 2011. AbaR4 replaces AbaR3 in a carbapenem-resistant *Acinetobacter* *baumannii* isolate belonging to global clone 1 from an Australian hospital. *J Antimicrob Chemother* 66(11):2484-91.

32. Nigro SJ, Hall RM. 2012. Antibiotic resistance islands in A320 (RUH134), the reference strain for *Acinetobacter baumannii* global clone 2. *J Antimicrob Chemother* 67(2):335-8.

33. Leverstein-van Hall M, Paauw A, Box A, Blok H, Verhoef J, Fluit A. 2002. Presence of integron-associated resistance in the community is widespread and contributes to multidrug resistance in the hospital. *J Clin Microbiol* 40(8):3038-40.

34. Gebreyes WA, Altier C. 2002. Molecular characterization of multidrug-resistant *Salmonella enterica subsp. enterica* serovar Typhimurium isolates from swine. *J Clin Microbiol* 40(8):2813-22.

35. Ng L-K, Martin I, Alfa M, Mulvey M. 2001. Multiplex PCR for the detection of tetracycline resistant genes. *Mol Cell Probes* 15(4):209-15.

32

36. Woodford N, Ellington MJ, Coelho JM, Turton JF, Ward ME, Brown S, Amyes SG, Livermore DM. 2006. Multiplex PCR for genes encoding prevalent OXA carbapenemases in *Acinetobacter* spp. *Int J Antimicrob Agents* 27(4):351-3.

37. Corvec S, Poirel L, Naas T, Drugeon H, Nordmann P. 2007. Genetics and expression of the carbapenem-hydrolyzing oxacillinase gene *bla*_OXA-23_ in *Acinetobacter baumannii*. *Antimicrob Agents Chemother* 51(4):1530-3.

38. Stokes H, Nesbø CL, Holley M, Bahl MI, Gillings MR, Boucher Y. 2006. Class 1 integrons potentially predating the association with Tn*402*-like transposition genes are present in a sediment microbial community. *J Bacteriol* 188(16):5722-30.

39. Nigro SJ, Post V, Hall RM. 2011. Aminoglycoside resistance in multiply antibiotic-resistant *Acinetobacter baumannii* belonging to global clone 2 from Australian hospitals. *J Antimicrob Chemother* 66(7):1504-9

**Table S6**. The primers used for sequencing by primer walking strategy

| **Primer** | **Sequence (5'-3')** | **Reference** |
| --- | --- | --- |
| RH2001 | GGAGTTGGTTTTGGTACAGCA | (8) |
| RH2010 | TTTCGTGACACTCTCGCTTG | (8) |
| F1 | AGTGCGGCCCCGGCTGTCGACG | This study |
| R1 | GATAACATCAACGCGCGGCAGC | This study |
| F2 | TATTCGCCAACAAGCTCTCAG | This study |
| R2 | TAATGCAGTGGCTGATGGGCG | This study |
